# Modulating the unfolded protein response with ONC201 to impact on radiation response in prostate cancer cells

**DOI:** 10.1038/s41598-021-83215-y

**Published:** 2021-02-19

**Authors:** Francesca Amoroso, Kimberley Glass, Reema Singh, Francisco Liberal, Rebecca E. Steele, Sarah Maguire, Rohinton Tarapore, Joshua E. Allen, Sandra Van Schaeybroeck, Karl T. Butterworth, Kevin Prise, Joe M. O’Sullivan, Suneil Jain, David J. Waugh, Ian G. Mills

**Affiliations:** 1 Patrick G Johnston Centre for Cancer Research, Queen’s University Belfast, Belfast, BT9 7AE UK; 2 Faculdade de Ciências e Tecnologia, Universidade Nova de Lisboa, 2825-516 Lisbon, Portugal; 3 Breast Cancer Now Toby Robins Research Centre, The Institute of Cancer Research, London, SW3 6JB UK; 4 Research and Development, Oncoceutics Inc., Philadelphia, PA 19104 USA; 5 The Northern Ireland Cancer Centre, Belfast City Hospital, Belfast, BT9 7AB UK; 6 Queensland University of Technology, Brisbane City, QLD 4000 Australia; 7 Nuffield Department of Surgical Sciences, University of Oxford, John Radcliffe Hospital, Oxford, OX3 9DU UK; 8 Centre for Cancer Biomarkers, University of Bergen, 5021 Bergen, Norway; 9 Department of Clinical Science, University of Bergen, 5021 Bergen, Norway

**Keywords:** Oncology, Cancer, Urological cancer, Prostate cancer, Cancer, Urological cancer, Prostate cancer

## Abstract

Prostate cancer (PCa) is the most common non-cutaneous cancer in men and a notable cause of cancer mortality when it metastasises. The unfolded protein response (UPR) can be cytoprotective but when acutely activated can lead to cell death. In this study, we sought to enhance the acute activation of the UPR using radiation and ONC201, an UPR activator. Treating PCa cells with ONC201 quickly increased the expression of all the key regulators of the UPR and reduced the oxidative phosphorylation, with cell death occurring 72 h later. We exploited this time lag to sensitize prostate cancer cells to radiation through short-term treatment with ONC201. To understand how priming occurred, we performed RNA-Seq analysis and found that ONC201 suppressed the expression of cell cycle and DNA repair factors. In conclusion, we have shown that ONC201 can prime enhanced radiation response.

## Introduction

Prostate cancer (PCa) is the most common cancer diagnosed in men and the second most common cause of cancer death after lung cancer. According to recent projections, prostate cancer incidence rates are predict to rise by 12% in the UK between 2014 and 2035, to 233 cases per 100,000 males by 2035^1^. Clinically localised PCa is treated using radical prostatectomy or radiotherapy to remove or destroy the cancer cells confined within the prostate capsule. However, 10–15% of the patients are diagnosed after their cancer has spread and present with advanced or inoperable disease^2^.

The prostate is a specialized accessory gland with a high secretory capacity. During cancer progression, cells experience mitogenic pressure and intracellular stress (e.g., metabolic pressure to rapidly grow and divide), detected by the endoplasmic reticulum (ER) as an accumulation of misfolded proteins. When the cells are not able to cope with the overload, the unfolded proteins accumulated in the ER, trigger an adaptive response called the Unfolded Protein Response (UPR)^3^. Attempting to clear the unfolded proteins and increase the capacity of the ER, the UPR activates several molecular pathways. Here, the so-called ER stress sensors PERK, IRE1alpha and ATF6 play a central role in the initiation and regulation of the UPR^4–7^. Previously, several studies have reported the activation of the UPR during tumour transformation and progression, leading to the acquisition of adaptive phenotypes to restricted nutrient supplies and therapies^8^. Upstream elements like XBP1 and ATF6 are upregulated in hepatocellular carcinomas^9^, in a range of breast cancer cell lines^10^, colon cancer and melanoma^8^. Although the UPR is generally viewed as a cytoprotective response, prolonged ER stress can directly regulate the cell death machinery through the activation of CHOP^11,12^. One of the mechanisms by which CHOP promotes apoptosis involves its ability to decrease anti-apoptotic Bcl-2 levels and stimulate the release of cytochrome C into the cytosol, resulting in the activation of apoptotic caspase 3^13^. Being a major secretory organ, the prostate is particularly reliant on the proper functioning of the ER and is vulnerable to agents or conditions that cause ER stress. Several studies have pointed to a positive association between ER/UPR markers and the development of prostate cancer^14^. Here, the activation of the IRE1alpha-XBP1 axis of the UPR contributes to tumorigenesis in contexts in which the driver is the androgen receptor^15^. Moreover in prostate cancers characterised by c-Myc overexpression and PTEN mutations, the ATF4 axis of the UPR plays a pro-survival role^16^. Even though the activation of the UPR is known to be the result of the accumulation of unfolded proteins in the ER^14^, treatments such as radiotherapy also increase stress and perturb general cellular homeostasis.

Radiotherapy causes double-strand DNA damage^17–19^. If unresolved, the radiation-induced DNA damage can lead to the production and the accumulation of unfolded and/or misfolded proteins in the ER^20^. Recent literature has shown that radiation exposure of glioblastoma stem cells activates key components of the UPR culminating in the autophagosome formation^21^. Moreover, the overexpression of UPR genes encoding GRP78 (BiP) and GRP94 has been extensively associated with radio-resistance in multiple cancer types, including breast, pancreatic and gastric cancers^22–24^. Recently, Drake and co-workers demonstrated that therapeutic doses of radiotherapy led to an upregulation of GRP78 in 72% of colorectal cancer cases receiving treatment^25^. Taking all these evidence together, targeting the UPR may provide an opportunity to enhance responses to radiotherapy.

ONC201 is an inhibitor of the dopamine receptors DRD2/3 and has previously been reported to induce apoptosis in haematological malignancies and solid tumours^26^. Pre-clinical studies have shown that ONC201 has an excellent safety profile at doses that exceed effective doses by tenfold and specificity for tumour versus normal cells in vitro^27^. The lack of cytotoxicity in normal cells was also confirmed in a panel of normal human bone marrow samples^28^. ONC201 was also shown to cause apoptosis in stem and progenitor AML cells and abrogated the engraftment of leukemic stem cells in vivo while sparing normal bone marrow cells^29^. In these models, ONC201 promoted apoptosis by increasing the translation of the transcription factor ATF4 in a non-canonical way, through an increase in the phosphorylation of eIF2α^29^. However, more recently Graves et al*.*, described a cytostatic effect mediated in Triple Negative Breast Cancer (TNBC) cells by the direct binding of ONC201 to the mitochondrial protease ClpP, which resolves into an alternative modulation of the integrated stress response pathway^30^. ONC201 has been also shown to be effective in early stage clinical trials in a number of cancer types^31,32^. Here we assess the impact of the ONC201 on all axes of the UPR to determine whether it can be used to enhance radiation response in PCa models.

## Results

### Chronic activation of the Unfolded Protein Response with imipridone ONC201 induces cell death by targeting the mitochondria

**Figure 1:**
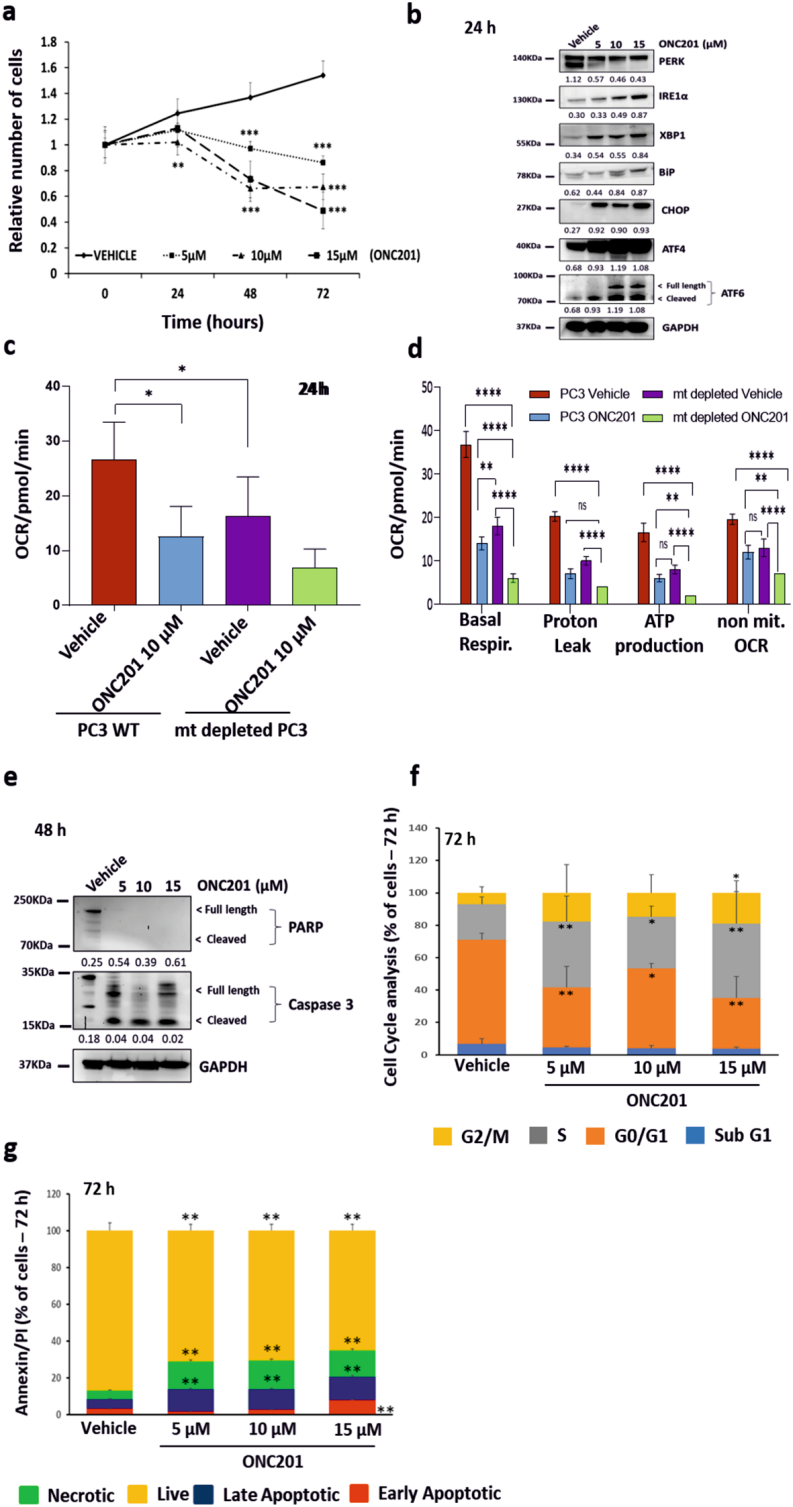
Chronic activation of the Unfolded Protein Response with ONC201 induces cell death by targeting the mitochondria. (**a**) Cell counts of PC3 cells treated with ONC201 (5, 10 and 15 µM) up to 72 h showed cytotoxic effects. (**b**) Western Blot analysis showing the overexpression of UPR related proteins in PC3 cells treated with ONC201 (5 µM, 10 µM and 15 µM) for 24 h, if compared to those treated with the Vehicle (representative of n = 3). (**c**) Measurement of the oxygen consumption rate (OCR) upon treatment of PC3-WT cells and mitochondrially depleted PC3 cells (mt depleted PC3) with Vehicle and ONC201 (10 µM) for 24 h. The measurements were taken after the addition of oligomycin (see Methods for details) and the results were normalised to cell counts post-treatment. (**d**) Measurements of the basal OCR, proton leak, ATP production and non-mitochondrial OCR upon treatment of PC3-WT cells and mt depleted PC3 with Vehicle and ONC201 (10 µM) for 24 h. (**c**,**d**) Representative traces of 3 independent experiments are shown in Supplementary Fig. 1d. (**e**) Western Blot analysis showing that ONC201 induced cell death trough the cleavage of Caspase 3 at 48 h in PC3 cells (representative of n = 3). (**f**) Cell cycle analysis of PC3 cells treated with ONC201 (5–15 µM) for 72 h showing that ONC201 induced cells to exit G0/G1 phase (ochre) to enter the S phase (grey). (**g**) AnnexinV/PI Assay on PC3 cells treated with ONC201 (5–15 µM) for 72 h showed ONC201 inducing cell death by both apoptosis (blue and red) and necrosis (green). One-way ANOVA test has been run comparing treated vs Vehicle on n = 3 experiments, *p ≤ 0.05; **p < 0.01; ***p < 0.001 (± SD). (**b**,**e**) The membranes were cut before the incubation with the primary antibody (see Material and Methods for specifications) and either detected alone or together. All the membranes were exposed for 3 min, with the exception of ATF4 and ATF6 (exposed for 6 min), PERK and Tubulin (exposed for 2 min). The corresponding densitometric analysis are in Supplementary Fig. 11a. Here the average is expressed as a number embedded within the figure (the SD are within the 5% acceptance of statistical significance).

The activation of the Unfolded Protein Response (UPR) is known to be the result of the accumulation of unfolded or misfolded proteins in the ER^14^. Among all the components of the UPR, CHOP is known to be a pro-apoptotic transcription factor expressed in response to acute stress, including the genotoxic stress that can be induced by therapies in cancer cells. In order to further evaluate the role played by the UPR in modulating the cellular response to therapy-induced stress in prostate cancer cells, we assessed the impact of ONC201, a small molecule known to activate the UPR in other models^26,33^. The treatment of PC3 cells, a cell line known to be radiation-resistant^34^, with ONC201 (5, 10 and 15 µM^35^) significantly induced cell death at 72 h (Fig. 1a). Interestingly, the cytotoxic effect observed with ONC201 at 72 h occurred after an early increase in the expression of all the main components of the UPR at 24 h (Fig. 1b; Supplementary Fig. 11a). This occurred despite any significant inhibition of Akt phosphorylation and despite the fact that PC3 cells express very low levels of DRD2, a reported molecular target of ONC201 (Supplementary Fig. 10b,c—associated full-length blots in Supplementary Fig. 12). It has been recently reported that ONC201 can induce cellular stress by disrupting mitochondrial function^30,35^. To further test this in the PC3 cell line, we depleted the mitochondria from our PC3-WT to generate a new cell line (mt depleted PC3 in the figures, Supplementary Fig. 1a–c). The mitochondrially depleted PC3 cells we generated showed a 95% reduction in mitochondrial DNA content, accompanied by a lower basal and maximal respiration rates—by about 80%—and reduced ATP production (green bars versus red bars). Using the Seahorse Assay, we observed that the activation of the UPR in response to ONC201 treatment at 24 h was accompanied by the significant inhibition of mitochondrial respiration (Fig. 1c,d and Supplementary Fig. 1d). ONC201 (10 µM) treatment reduced the oxygen consumption rate (OCR) by about 50% (Fig. 1c—blue bar versus red bar) and the principal respiratory parameters (basal OCR, the proton leak and the ATP production) were all reduced by about 60% (Fig. 1d—blue bars versus red bars). The significant reduction that we observed in mitochondrial activity with ONC201 treatment (approximately 50%) was therefore of a similar order of magnitude to that achieved by mitochondrial depletion in this cell-line (Fig. 1c,d—purple bars versus blue bars, Supplementary Fig. 1d). We observed a small further reduction when we treated the mitochondrially depleted PC3 cells with ONC201 (10 µM) (Fig. 1c,d—green bars, Supplementary Fig. 1d). In order to further establish whether effects on mitochondrial functions were a major feature of ONC201 treatment, we cultured cells in high glucose (4.5 g/l glucose) condition to enhance their metabolic capacity. In so doing, we were able to overcome the inhibitory effects of ONC201 on respiration (Supplementary Fig. 1f–h).

Cytotoxicity induced by a long-term exposure to ONC201 arose from the Caspase 3 activation at 48 h (Fig. 1e; Supplementary Fig. 11a). Cell cycle analysis at 72 h after the administration of ONC201 (Fig. 1f) showed a significant increase in S phase cells, accounting for about 30% of the surviving population (grey bars) and also a significant increase in G2/M phase cells, accounting for about 20% of the surviving population (yellow bars). There is evidence that ONC201 modulates the cell cycle in haematological malignancies and leukemic stem/progenitor cells^36^. In this setting, ONC201 causes p53-independent apoptosis and a delayed S phase transition^36^. At 72 h we also observed a significant and dose-dependent increase in apoptosis (blue and red bars) and necrosis (green bars) affecting 20% of the cell population (Fig. 1g).

### Priming Prostate Cancer cells with ONC201 increases the efficacy of irradiation

**Figure 2:**
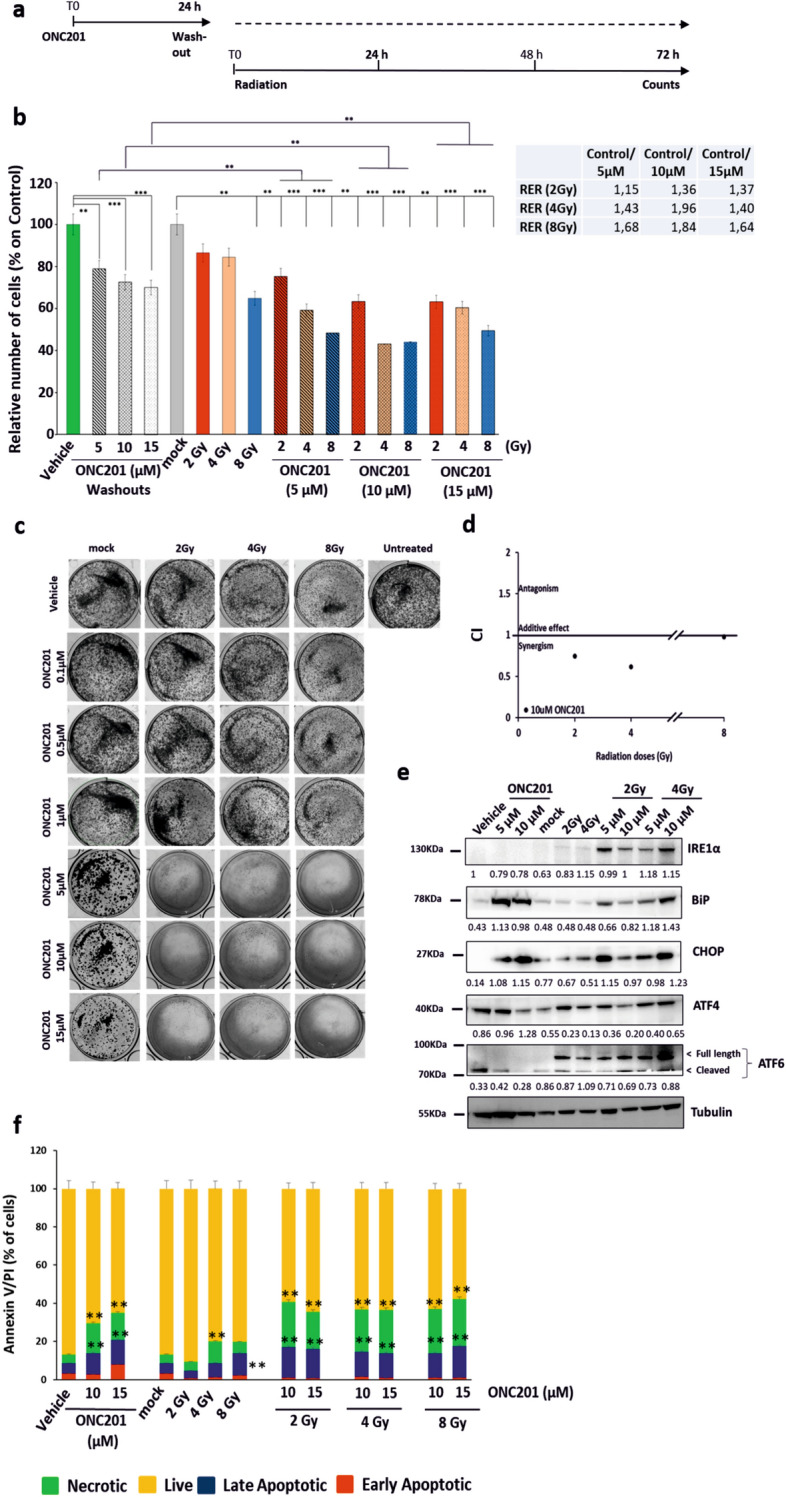
Priming Prostate Cancer cells with ONC201 increases the efficacy of irradiation (**a**) Schematic representation of the treatment in which PC3 cells have been primed with ONC201 before being radiated with single doses of Xrad. (**b**) Cell counts showed that priming PC3 cells to radiation (Xrad) with ONC201 for 24 h impacts cells survival at 72 h. Radiation Enhancement Ratio (RER) is shown for ONC201 (5–15 µM) and Radiation (2, 4 and 8 Gy) and calculated out of 3 independent experiments. (**c**) Clonogenic Assay. Priming PC3 cells to radiation (2, 4 and 8 Gy) with ONC201 (0.1–15 µM) for 24 h prevents colony formation for concentrations higher than 1 µM. The cells were treated with ONC201 for 24 h and then either mock irradiated or irradiated with the indicated doses. Post-treatment, the cells were cultured in clonogenic conditions and photographed after 1 week (Images representative of n = 3). (**d**) ONC201 10 µM has a synergistic effect with all doses of radiation (2, 4 and 8 Gy) at 72 h (CompuSyn). (**e**) Western Blot analysis of the expression of the main components of the UPR upon priming PC3 cells to radiation (Xrad) with ONC201 for 24 h. Samples were harvested at 72 h from the irradiation (Representative of n = 3). Densitometric analysis of n = 3 WB are shown in Supplementary Fig. 2c. Here the average is expressed as a number embedded within the figure (the SD are within the 5% acceptance of statistical significance).The membranes were cut before the incubation with the primary antibody (see “Materials and methods” for specifications) and either detected alone or together. All the membranes were exposed for 3 min, with the exception of ATF4 and ATF6 (exposed for 6 min), PERK and Tubulin (exposed for 2 min). (**f**) Cell death analysis through Annaxin V/PI detection of PC3 treated with ONC201 (5 and 10 µM) and Radiation (as described in (**a**)). Samples were harvested at 72 h. One-way ANOVA test has been run comparing treated vs Vehicle and No Radiation on n = 3 experiments, *p ≤ 0.05; **p < 0.01; ***p < 0.001 (± SD).

Having observed a clear temporal progression from UPR activation and mitochondrial inhibition at 24 h to cell death at 72 h, we sought to test whether short-term administration of ONC201 could prime subsequent cell death responses to irradiation (Fig. 2a). We used 3 concentrations of ONC201 (5, 10 and 15 µM) and 3 doses of radiation (2, 4 and 8 Gy) (Fig. 2b). After 24 h the compound was washed out and the cells were irradiated. At 72 h post-irradiation, we observed 30–50% more cell death in conditions in which ONC201 pre-treatment has been used than with irradiation alone. To determine whether this was indeed a radiation–sensitisation effect resulting from ONC201 pre-treatment, we calculated the Radiation Enhancement Ratio (RER) at 72 h post-irradiation (Fig. 2b, right side). The RER was calculated by dividing the radiation dose necessary to induce cell death by 10% after radiation alone by the radiation dose necessary to induce cell death by 10% after sequential treatment (with a RER value of > 1 being indicative for radio-sensitization)^37^. The RER values following ONC201 pre-treatment were all > 1 for all concentrations used (5–15 µM) indicating a significant radio-sensitization. The ratio was higher for the 10 µM dose than for the 5 µM dose and greater when 4 Gy irradiation was used rather than 2 Gy. The ratio arising from 15 µM pre-treatment was lower than at 10 µM and this may reflect off-target effects at this higher dose. The reported GI50 for ONC201 is in the range 1–10 µM^38,39^. Clonogenic assays are routinely used to characterise the radiation response of cells to sensitizers and typically run for 1–2 weeks post-treatment. ONC201 treatment (5, 10 and 15 µM doses) was followed by significant radiation-induced cell death and consequently no colonies were visible in a clonogenic assay when 15 µM was used compared to radiation-alone (Fig. 2c). To address this we therefore expanded the dose range to include lower ONC201 concentrations (0.1 µM, 0.5 µM and 1 µM Fig. 2c). Treating cells with 1 µM ONC201 followed by 8 Gy irradiation reduced colony formation versus 8 Gy irradiation alone. Lower concentrations did not show clear qualitative differences in clonogenicity when comparing vehicle to ONC201 pre-treatment. This suggests that ONC201 pre-treatment may sensitise cells to radiation in a narrow dose range when clonogenic potential is used as a readout and that enhanced cytotoxicity upon ONC201 pre-treatment dominates at 5, 10 and 15 µM concentrations.

We then confirmed that pre-treatment with ONC201 (10 µM) was indeed synergistic with radiation (Fig. 2d). Blotting cell lysates, we confirmed that ONC201 led to sustained increases in the expression of all the components of the UPR and particularly of BiP, IRE1alpha, XBP1, ATF6, ATF4 and CHOP at 24 h (Supplementary Fig. 2a—left side and b). At 72 h, more intriguingly, distinct components of the UPR still remained upregulated depending on the treatment. More specifically, the ONC201 treatment led to increases in the total expression of PERK, BiP, ATF4 and CHOP whereas radiation led to consistent increases in IRE1α and ATF6 levels upon treatment with 4 Gy (Fig. 2e; Supplementary Fig. 2a—right side and c). Priming prostate cancer cells with ONC201 for 24 h followed by radiation, maintained the over-expression of both signatures at 72 h (PERK/CHOP plus IRE1a/ATF6) (Fig. 2e; Supplementary Fig. 2a—right side and c). Given that the treatment with ONC201 followed by radiation was inducing the overexpression of proteins of the UPR known to promote cell death, we sought to determine how the cells were dying. Once again, we observed very distinct responses when the cells were treated with radiation alone or ONC201. Radiation induced cell death primarily by apoptosis (ca. 10% of the cells) and by contrast ONC201 induced necrotic cell death in up to the 20% of the cells. The overall effect of the sequential treatment on cell survival was the activation of both necrosis (green, up to 30%) and apoptosis (red and blue, up to 20% of the total population), combining the cell death characteristics of each individual treatment (Fig. 2f).

### RNA-Seq analysis reveals that ONC201 restricts the expression of cell cycle progression and DNA repair pathway genes

**Figure 3:**
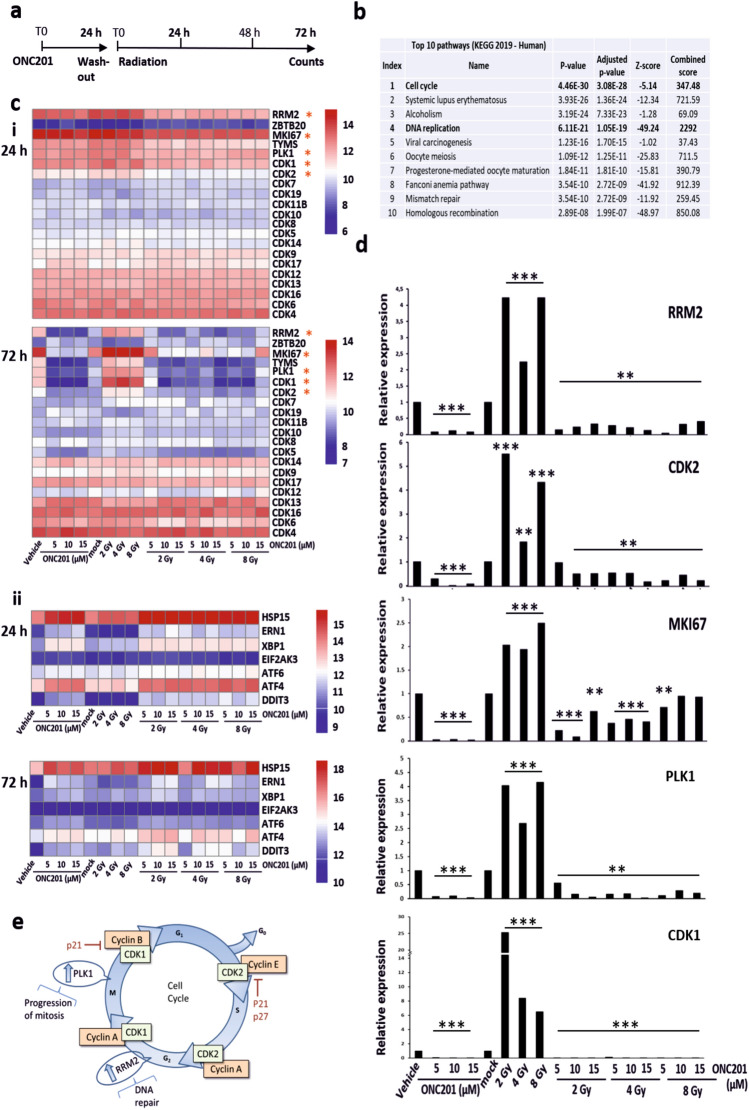
RNA-Seq analysis reveals that ONC201 restricts the expression of cell cycle progression and DNA repair pathway genes. (**a**) Schematic representation of the treatment in which PC3 cells have been primed with ONC201 before being radiated with single doses of Xrad (CorelDRAW Graphics Suite 2019, 64-Bit). (**b**) Pathway analysis of our genes of interest in KEGG Human 2019 pathway database, after RNA-seq analysis of PC3 cells treated as shown in (**a**), revealed that the most impacted pathway is cell cycle related (iEnrichr web tool^66,67^). (**c**) Heatmaps analysis of RNAseq data (log2 of gene counts) of PC3 cells treated as shown in (**a**). Cells were harvested at 24 h (**c**, i and ii top) and 72 h after the irradiation (**c**, i and ii bottom). The genes listed are the most differentially regulated (see Methods for details on the analysis with RStudio software, Version 1.2.5033, release name "Orange Blossom" (RStudio Team (2020). RStudio: Integrated Development for R. RStudio, PBC, Boston, MA URL http://www.rstudio.com/). (**d**) Validation through TR-PCR of the five most relevant and differentially regulated genes at 72 h from the final radiation dose (RRM2, CDK2, PLK1, MKI67, CDK1). One-way ANOVA test has been run comparing treated vs Vehicle and No Radiation on n = 3 experiments, **p < 0.01; ***p < 0.001 (± SD). (**e**) Schematic representation of the cell cycle check points (RRM2, CDK2, PLK1, MIK67, CDK1) were drawn utilizing CorelDRAW Graphics Suite (CorelDRAW Graphics Suite 2019, 64-Bit).

To reveal more about the molecular mechanisms through which ONC201 may function as a radio-sensitizer, we performed RNA-Seq analysis on treated PC3 cells (Fig. 3a). Priming PC3 cells to radiation utilizing ONC201 for 24 h (then washed out) and collecting the cells at 24 h and 72 h after irradiation, we observed that through the principal component analysis (PCA) our samples clustered according to the timepoints of harvesting (Supplementary Fig. 3a—red dots vs blue dots respectively). To identify significant differentially expressed genes, we therefore focussed on a single timepoint, 72 h, post-irradiation and on genes that were significantly overexpressed in surviving cells (Table 1). Pathway analysis (Fig. 3b; Supplementary Fig. 3b and Table 2) revealed enrichment for cell cycle progression, DNA replication and repair related pathways^40–42^. Taking all this in consideration, we generated heatmaps for the genes that, at 72 h, were most significantly upregulated in cells surviving radiation (red squares) and downregulated by ONC201 treatment (blue squares) (Fig. 3c). The most impacted genes were RRM2, MKI67, TYMS, PLK1, CDK1 and CDK2 (Fig. 3c,i—72 h). These same genes appeared to be upregulated by both ONC201 and radiation at 24 h together with other cell cycle regulators (Fig. 3c,i—24 h). By contrast the expression of CDK4, CDK6, CDK12, CDK13 and CDK16 were not significantly affected by ONC201 treatment at either timepoint, indicating some selectivity in the effects of the drug on cyclin-dependent kinases. RNA-Seq data also confirmed that ONC201, both in single treatment and as a radio-sensitizer, increased the expression of genes encoding ATF4 and CHOP at 24 h (Fig. 3c,ii—24 h). In contrast to the increased expression of UPR-related proteins we observed at 72 h, the transcript levels of factors such as CHOP and ATF4 were downregulated at 72 h in our RNA-Seq data (Fig. 3c,ii—72 h). The lack of correlation between the expression of UPR transcripts and proteins has been previously reported and may reflect partly an impact on the normal protein folding and turnover functions of cells experiencing stress^43,44^. This may also reflect feedback compensations for post-transcriptional defects. We then validated in vitro through RT-PCR the expression levels of transcripts encoding RRM2, MKI67, CDK2, CDK1, and PLK1 (Fig. 3d) based on their significant differential expression and regulatory roles in the control of cell cycle progression and DNA damage repair (as shown in Fig. 3e). Three of these transcripts encode proteins that are known to be druggable (PLK1, CDK1, CDK2) and are key cell cycle regulators. We further investigated the impact of ONC201 on the expression of these factors and confirmed by Western blotting that polo-like kinase 1 (PLK1) was downregulated at the protein level by ONC201, despite being overexpressed when cells were treated with radiation alone (Supplementary Fig. 5a). Among the genes we validated in vitro, we also confirmed the expression levels of MKI67 because coding for a protein (Ki-67) commonly used as a marker of proliferation, thus corroborating the phenotypic data we described previously.

**Table 1:** Table with a list of the most differentially expressed genes shortlisted from the RNA-Seq analysis (Ratio > 1.5 on Vehicle-treated samples) in relation to their direct regulation of the most effected pathways (Enrichr).

"Reactome_Cell Cycle"	"Reactome_Cell Cycle" and "Reactome_RHO effectors"	"Reactome_Cell Cycle" and "Reactome_DNArepair"	"Reactome_RHO effectors"	"Reactome_DNArepair"
CDK1	PLK1	FEN1	PRC1	XRCC3
TYMS	INCENP	PCNA	KIF14	KPNA2
RRM2	MAD2L1	RFC3	CIT	CLSPN
LMNB1	CDCA8	CDK2		DTL
KIF20A	KIF2C			FANCI
CDCA5	SPC24			FANCD2
MYBL2	BIRC5			NEIL2
SKP2	SGOL2			POLH
AURKA	CASC5			POLM
KIF23	NDC80			TIMELESS
UBE2C	BUB1			POLQ
NCAPD2	BUB1B			TDP1
NUP210	DSN1			RAD18
HAUS8	CENPE			
TOP2A	CENPC			
CCNB2	CENPF			
CCNB1	CDC20			
MCM8	ZWINT			
MCM4	NUP37			
TMPO	KNTC1			
TPX2				
NCAPH				
PTTG1				
LPIN1				
NCAPG				
POLA2				
NCAPD3				
DBF4				
SMC2				
MCM6				
NCAPG2				
TUBB4B

**Table 2:** Table summarizing the top three most impacted pathways by the most differentially expressed genes obtained through RNA-Seq analysis (Ratio > 1.5 on Vehicle-treated samples), according to the Reactome tool.

Top 3 pathways (Reactome)		
Reactome pathway	Overlap	Adjusted p-value
Cell Cycle	61/462	7 x 10^−48^
RHO GTPase Effectors	28/255	4 x 10^−19^
DNA Repair	22/285	7 x 10^−12^

### ONC201 induces an expansion of the S phase of the cell cycle

**Figure 4:**
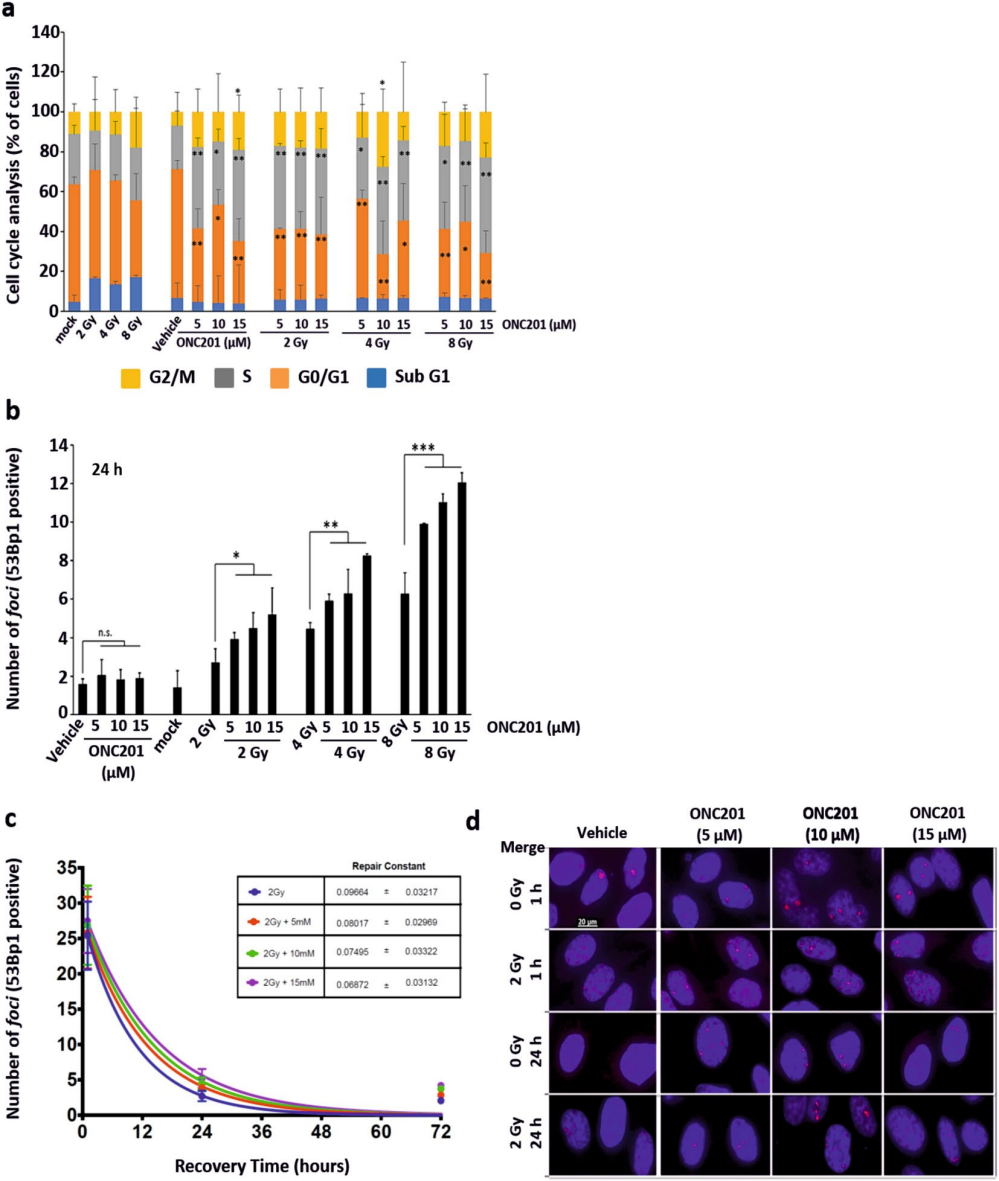
ONC201 determines the accumulation of *foci* into the nuclei of cells primed to radiation. (**a**) Cell cycle analysis of PC3 cells primed to radiation (Xrad) with ONC201 (5–15 µM) for 24 h showed an expansion of the cell population in S (grey) and G2/M (yellow) phases (n = 3). (**b**) Number of 53Bp1^+^ *foci* per cell at 24 h post-irradiation in PC3 cells. Samples were analysed at the time points schematically represented in Fig. 3a (n = 3). (**c**) Analysis of the kinetics of repair from the DNA damage induced by radiation in PC3 cells (n = 3). Samples were treated and analysed at the time points schematically represented in Fig. 3a. (**d**) Immunofluorescence analysis (Merge) of the *foci* formation, determined through 53Bp1 (red) staining at 1 and 24 h from radiation (representative of n = 3). The total number of *foci* counted is represented in panel (**b**). One-way ANOVA test has been run. The data shown in panel (**b**) have been run through ANOVA test on Ranks and further analysed with Dunnett’s Method. *p ≤ 0.05; **p < 0.01; ***p < 0.001 (± SD).

To further investigate the role of ONC201 in modulating genes controlling the cell cycle progression and the DNA damage response, we performed cell cycle analysis on cells treated and collected at 72 h (Fig. 4a). We observed a doubling of the population in S phase (grey bars) and an expansion of the population in G2/M phase (up to 20% more, yellow bars) upon treatment with ONC201 alone**.** These increases were even greater when the radiation was primed by ONC201. Thus, we hypothesised that the impact of ONC201 as a radio-sensitizer was achieved also by activating the pro-death branches of the UPR and restricting both the cell cycle progression and the DNA damage repair machinery in surviving cells. This hypothesis was confirmed when we analysed the nuclear localization of 53Bp1 by quantitating *foci* positively stained with this marker (Fig. 4b–d; Supplementary Fig. 4). The number of 53Bp1-positive *foci* detected at 24 h post-irradiation increased proportionally to the priming concentration of ONC201 to which the cells have been exposed and was sixfold greater with the highest doses (Fig. 4b). This led us to conclude that PC3 cells pre-treated with ONC201 were accumulating more DNA damage for a given dose of radiation, due to inhibitory effects on the expression of proteins required to resolve radiation-induced DNA damage and promote cell cycle re-entry. This pattern was maintained at a 72-h timepoint post-irradiation, thus confirming that pre-treatment with ONC201 impaired the resolution of the DNA damage post-irradiation (Fig. 4d; Supplementary Fig. 4a).

**Figure 5:**
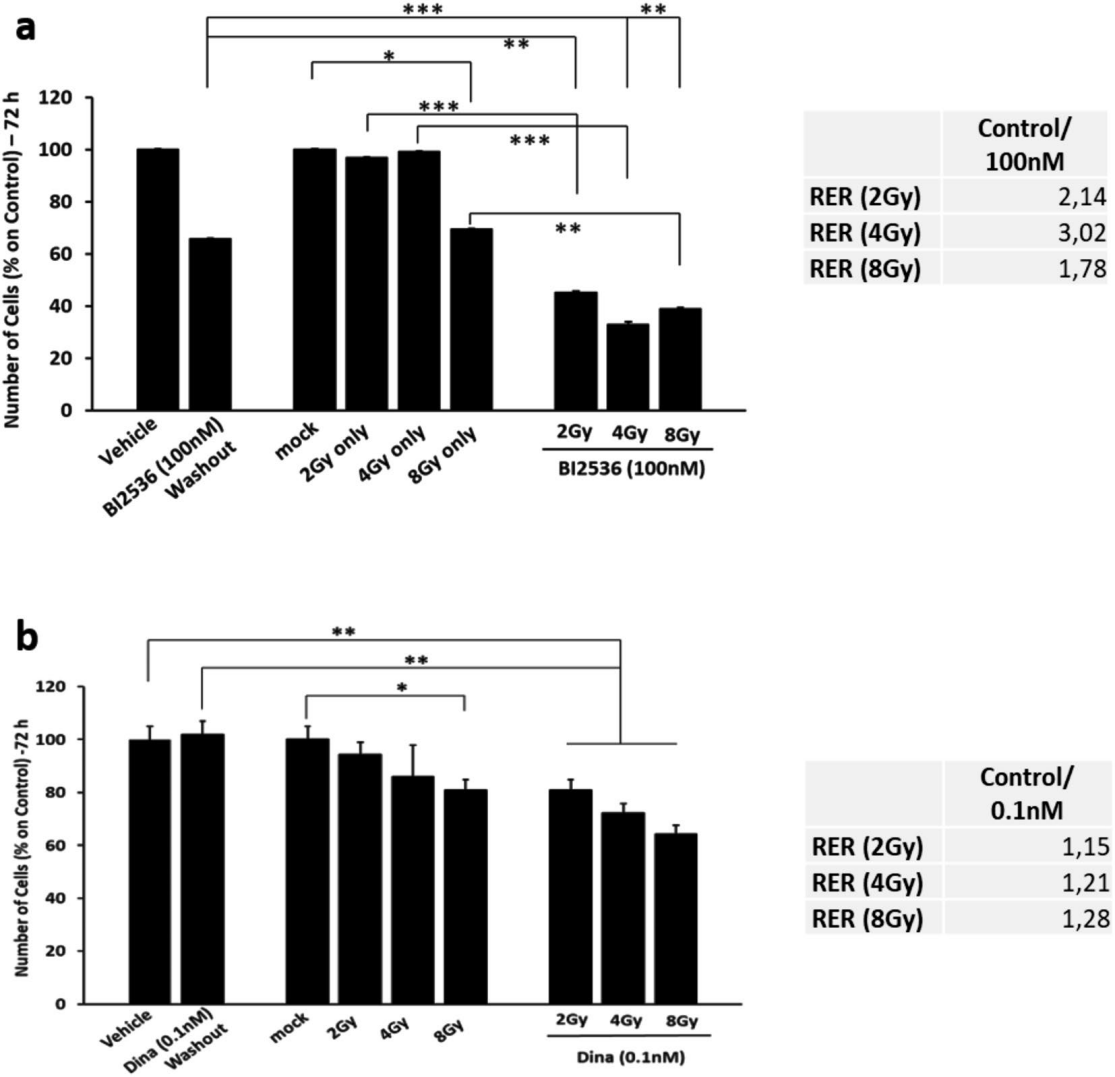
Pre-treating PC3 cells with PLK1 and CDK inhibitors for 24 h prior to radiation, enhances in cell death. (**a**) Cell counts of PC3 primed to radiation (Xrad) with the PLK1 inhibitor BI2536 (100 nM) for 24 h. Counts were taken at 72 h from the radiation (2, 4 and 8 Gy). Radiation Enhancement Ratio (RER) is shown for BI2536 (100 nM) and Radiation (2, 4 and 8 Gy) and calculated out of 3 independent experiments. (**b**) Cell counts of PC3 primed to radiation (Xrad) with a sub-toxic concentration of the CDKs inhibitor Dinaciclib for 24 h (0.1 nM). Radiation Enhancement Ratio (RER) is shown for Dinaciclib (0.1 nM) and Radiation (2, 4 and 8 Gy) and calculated out of 3 independent experiments. One-way ANOVA test has been run comparing treated vs Vehicle and mock radiated on n = 3 experiments, *p ≤ 0.05; **p < 0.01; ***p < 0.001 (± SD).

Based on these findings, we hypothesized that drugs that could restrict cell cycle progression by directly inhibiting PLK1 or other cell cycle regulators might achieve similar sensitising effects if administered prior to radiation. We therefore treated PC3 cells with the PLK1 inhibitor BI2536 (100 nM)^45^ (Fig. 5a). Pre-treating PC3 cells with this drug for 24 h prior to radiation, in a similar manner to ONC201, led to a 50% enhancement in cell death. This is a more significant sensitization than we observed with the highest pre-treatment dose of ONC201. We also observed the same expansion that we observed with the ONC201 pre-treatment in the proportion of cells in S phase (grey bars) and in the G2/M transition (yellow bars, Supplementary Fig. 5b), as well as increases in cell death by apoptosis and necrosis of around 20% (Supplementary Fig. 5c). We also calculated the Radiation Enhancement Ratio (RER) to assess the impact of pre-treating cells with BI2536 on subsequent responses to radiation. At 72 h post-irradiation BI2536 increased the efficacy by three-fold when administering a 4 Gy dose (Fig. 5a—right side).

Another cell cycle check point gene validated in vitro from the RNA-Seq analysis was CDK2 (Fig. 3c,d). CDK2 was downregulated by ONC201 at the mRNA level and upregulated by radiation alone (Fig. 3d). To assess whether this too could be exploited as a radiation sensitizer, we pre-treated PC3 cells with a sub toxic dose of Dinaciclib, a CDK inhibitor (0.1 nM) (Fig. 5b). By doing so, we were able to significantly reduce the cell number by up to 35% at 72 h and enhanced responses were once again confirmed by calculating the RER (Fig. 5b—right side). We also observed the same expansion in the proportion of cells in S phase (grey bars) and at the G2/M transition (yellow bars) that we had observed by pre-treating with ONC201, as well as increased cell death by apoptosis and necrosis of ca. 20% (Supplementary Fig. 6a,b).

To determine whether treating with ONC201 before the irradiation is more effective than the vice versa, we subjected PC3 cells that had survived hypo-fractionated doses of radiation (5 times 2 Gy and 10 times 2 Gy) to ONC201 and found that prior exposure to radiation led to acquired resistance to ONC201 (Supplementary Fig. 5d,e).

### ONC201 pre-treatment enhances radiation response in other cancer types

**Figure 6:**
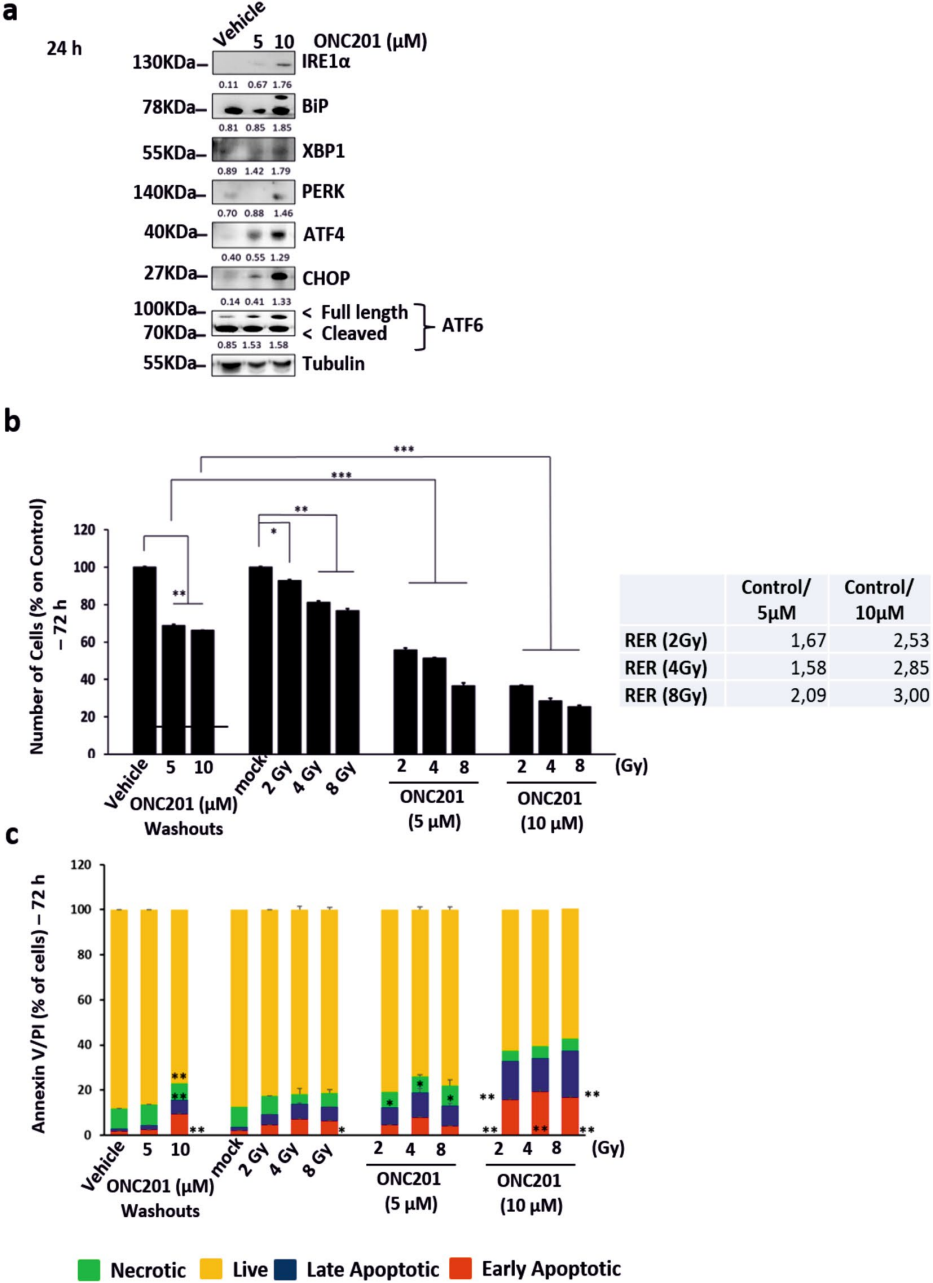
ONC201 pre-treatment enhances radiation response in other cancer types. (**a**) Western Blot analysis showing the overexpression of UPR related proteins in colorectal cancer cells HT-29 treated with ONC201 (5 and 10 µM) for 24 h, if compared to those treated with the Vehicle (representative of n = 3). The corresponding densitometric analysis are in Supplementary Fig. 11b. Here the average is expressed as a number embedded within the figure (the SD are within the 5% acceptance of statistical significance). The membranes were cut before the incubation with the primary antibody (see “Materials and methods” for specifications) and either detected alone or together. All the membranes were exposed for 3 min, with the exception of XBP1 (exposed for 7 min) and Tubulin (exposed for 2 min). (**b**) Cell counts of HT-29 colorectal cells primed to radiation (Xrad, 2- 8 Gy) with ONC201 for 24 h showed ONC201 impacting cells survival at 72 h. Radiation Enhancement Ratio (RER) was calculated for ONC201 (5, 10 µM) and Radiation (2, 4 and 8 Gy) out of 3 independent experiments. (**c**) Cell death analysis through Annaxin V/PI detection of HT-29 cells treated with ONC201 (5 and 10 µM) 24 h prior Radiation. Samples were harvested at 72 h. One-way ANOVA test has been run comparing treated vs Vehicle and mock radiated on n = 3 experiments, *p ≤ 0.05; **p < 0.01; ***p < 0.001 (± SD).

Given the sensitizing effects of ONC201 on PC3 prostate cancer cells to radiation (Fig. 2a–c), we wanted to assess whether ONC201 could have similar effects on other cell lines and cancer types. We therefore treated HT-29 cells, a colorectal cancer cell line, with ONC201 (5 and 10 µM) for 24 h and tested whether we were able to activate all the arms of the UPR (Fig. 6a; Supplementary Fig. 11b). ONC201 at a concentration of 10 µM increased the expression of proteins in all the arms of the UPR. We primed HT-29 cells, a colorectal cancer cell-line, to radiation with ONC201 (5 and 10 µM) for 24 h. We followed up with 3 doses of radiation (2, 4 and 8 Gy) (Fig. 6b). By doing so, we increased cell death by 50% (Fig. 6b) and enhanced apoptosis by 40% (Fig. 6c). The RER calculation confirmed this sensitisation effect showing once again a dose-dependent three-fold increase in efficacy when cells were pre-treated with ONC201 for 24 h (Fig. 6b—right side). Although the enhanced radiation response was observed both for the PC3 and HT-29 cells in this study, they express dramatically different levels of DRD2 which has been previously reported to be a target of ONC201 (Supplementary Fig. 10b—associated full length blots in Supplementary Fig. 12). HT-29 cells express high levels of this receptor whereas in PC3 cells it is barely detectable by Western blotting). Together these data indicate that these radio-sensitisation effects are robust and future studies will extend this work to additional models and further define the molecular basis for this effect.

## Discussion

Radiotherapy (brachytherapy, external beam radiation, and proton therapy) remains a highly effective modality in the treatment of multiple stages of prostate cancer. Between 10 and 15% of patients are diagnosed after their cancer has spread and/or after first doses of radiation [with and without androgen deprivation therapy (ADT)] fail, progressing to advanced or inoperable prostate cancer^2^. Understanding the molecular factors underpinning radiotherapy response and treatment failure is an area of research in which several signaling pathways have been recently implicated, including the UPR.

In this study, we have assessed whether enhancing the expression of UPR components in a sustained manner can synergise with radiation^46^.

ATF4 activation can have both pro-apoptotic and pro-survival effects, in particular supporting cell-intrinsic metabolic adaptations^47–50^. In fact, tumour cells inside a growing tumour mass, often overexpress ATF4 to alleviate the stress from rapid proliferation and limited nutrient supply. The crucial impact of ATF4 activation on cell viability has been linked to the ATF4-dependent modulation of the balance between direct expression of adaptive versus pro-apoptotic targets and indirect control on targets involved in autophagy and protein synthesis^51^. To verify our hypothesis we tested a new compound (ONC201) that was reported to induce an integrated stress response ATF4- and CHOP-dependent and in phase I and II trials in other cancer models^26,33^ (Fig. 1). We observed an ONC201-induced activation of all the arms of the UPR in the first 24 h. ONC201 increased cell death by inducing apoptosis and necrosis at 72 h from the treatment. Cell death is known to be triggered by unresolved UPR activation and autophagy, apoptosis has also been described through IRE1alpha-dependent activation of JNK pro-apoptotic pathways^52^. Necrosis, on the other hand, has been linked to a sustained activation of the anticipatory UPR initiated by ATP depletion in ER^+^ breast cancer cells^53^. ONC201 induced necrosis in our hands but also induced an expansion of the cell population in S phase. We found that ONC201 perturbs mitochondrial activity and reduced oxidative phosphorylation (Fig. 1c–e; Supplementary Fig. 2a–d). This finding accords with recent literature reporting ONC201 as an inhibitor of mitochondrial function and oxidative phosphorylation in cancer models of TNBC and glioblastoma^30,35,54^. In these models, it led to a substantial reduction in the expression of genes encoding mitochondrial proteins. Our data showed that when PC3 cells were cultured in high glucose they became unresponsive to ONC201 (Supplementary Fig. 1f–h). We therefore believe that the mitochondria of highly glycolytic cells might not be perturbed by ONC201. This might be true for hypoxic regions of cancers. To fully evaluate this, a further study will be required using tissue explants/ex vivo tissue culture and imaging. Hypoxic signalling has been associated with poor prognosis prostate cancers by us and others. In a genomic setting it is linked to the acquisition of copy number instability. Further evaluating the function of ONC201 in more complex tissue-based and in vivo prostate cancer models will therefore be critical to determining whether hypoxic regions are less affected by treatment or whether there are durable effects across the whole tumours.

Scheduling treatment is also central to achieving effective responses. We observed increased expression of the UPR components after 24 h of ONC201 treatment prior to the induction of cell death. Whilst this provided created an in vitro time window that we could exploit to enhance radiation responses following short-term ONC201 treatment (Fig. 2b). It will be important to establish whether this holds true in more complex pre-clinical models. If confirmed in more complex models and in a clinical setting, this could provide an opportunity to change treatment schedules to reduce dosing or the duration of treatment exposure and enhance treatment response whilst minimising toxicity.

Radiotherapy is known to cause double-strand DNA damage^17^. The majority of these DNA double-strand breaks (DSBs) can be repaired by non-homologous end-joining (NHEJ) through the whole cell cycle and by homologous recombination repair (HRR) during late S and G2 phases^55^. In both scenarios, histone alterations, nucleosome repositioning and changes in the higher-order folding of the chromatin fibre occur prior to the repair of lesions^56^. These modifications cause massive recruitment of the proteins gamma H2AX and 53Bp1 in large segments of the lesioned chromatin^57–59^. Because of these mechanisms, the S phase of the cell cycle is typically considered to be the phase that supports the emergence of radiation-resistant cells. In leukemic stem/progenitor cells ONC201 causes p53-independent apoptosis and a delayed S phase transition^36^. In our hands we also observed an S-phase expansion as well as increased cell death. The paradox of S-phase expansion, accompanied by less efficient DNA repair and, increased cell death was partly resolved by RNA-seq. This revealed that ONC201 pre-treatment suppressed the increased expression of cell cycle progression and DNA repair pathway genes associated with surviving cells treated solely with irradiation. PLK1 and CDK1, in particular, are both known regulators of the G2/M-DNA damage checkpoint. They ensure that cells don't initiate mitosis until damaged DNA or incompletely replicated DNA is sufficiently repaired after replication (which occurs during the S phase, or Interphase)^60^. Cells that have a defective G2/M checkpoint enter mitosis before repairing their DNA, leading to apoptosis or death after cell division^60^. By implication short-term pre-treatment of cells with inhibitors of these kinases should also therefore enhance radiation response and we confirmed this using BI2536 or Dinaciclib prior to radiation (Fig. 5) (Supplementary Figs. 5 and 6).

In conclusion, we have shown that short-term treatment with ONC201 prior to irradiation can enhance subsequent radiation responses. We suggest that this is because UPR activation can restrain the expression of cell cycle regulators and DNA repair pathway enzymes this restricting the ability of cells to resolve damage. The mechanism underpinning this relationship remains to be resolved. We suggest however that mitochondrial function affects this response to some extent. As this work is developed in more complex pre-clinical models, and ultimately clinically, it will be important to define the hypoxic and proliferative indices of treated tumours. These represent biologies that are most perturbed by ONC201 treatment in our studies and have been independently linked to poor prognosis prostate cancers. Consequently, they may well need to be accounted for in attempting to predict ONC201 treatment response.

## Material and methods

### Seahorse assay (analysis of mitostress parameters and metabolic flux)

Mitostress test analysis was performed in a Seahorse XFe96 instrument. The equal numbers of cells (10,000 cells/well) were seeded in a XFe96 culture microplate. They were cultured in standard media in 5% CO_2_ at 37 °C and treated with 10 µM ONC201 for 24 h. Mitostress analysis protocol was followed according to the manufacturers guidelines (Agilent). In brief, on the day of experiment prior to the run, culture media from the culture microplate was replaced with seahorse XF assay media (Agilent, California, USA) supplemented with 1 mM Pyruvate, 2 mM Glutamine and 10 mM glucose (bicarbonate free). Oxygen consumption rate (OCR) were measured at baseline, after addition of oligomycin 2 μM, carbonyl cyanide 4-(trifluoromethoxy) phenylhydrazone (FCCP) 2 μM and rotenone 0.5 μM. Data were normalised by cell numbers after the treatment. The metabolic and respiratory parameters were analysed in the wave software.

### Estimation of mitochondrial DNA content using Real-time PCR

Real time PCR reactions were used to quantify the mitochondrial DNA relative to nuclear DNA. The quantification of mitochondrial/nuclear DNA was performed as mentioned by JP Rooney et al.^61^. Total genomic DNA was isolated using DNeasy kit (Qiagen, Hilden, Germany). Primers (Eurofins Genomics) had the following sequences, forward: MitHu3130F, AGGACAAGAGAAATAAGGCC, reverse: MitHu3301R TAAGAAGAGGAATTGAACCTCTGACTGTAA for the mitochondrial fragment. Also, forward: APP137F TTTTTGTGTGCTCTCCCAGGTCT and reverse: APP210R TGGTCACTGGTTGGTTGGC for the nuclear fragments. Light cycler 480 SYBR Green Master I Roche was used as per manufacturer’s instructions. 1 ul of both reverse and forward primers from 100 nM stocks and 10 ng of target DNA was taken for per reaction. Thermal cycling conditions were 95 °C denaturation and enzyme activation 10 min and followed by 40 cycles of 95 °C denaturation for 15 s, 72 °C annealing for 60 s and 60 °C for 60 s.

### Proliferation and annexin V/PI assay

For the Proliferation assay, cells were treated as described in supplementary materials and maintained in culture up to 72 h from the administration of the treatment. When irradiated, for each experiment, unexposed controls were prepared and treated as sham exposures (mock) and harvested at matched time intervals. Adherent cells surviving radiation and/or drug administration where imaged in bright field (Leica CTR600, Leica, Wetzlar, Germany) at each time point and counted using ImageJ software (Public Domain, BSD-2). A minimum of 6 pics/well was taken as representative of the samples. Counts were normalized on corresponding T0. In the case of sequential experiments in which pre-treatments with ONC201, BI2536 and Dinaciclib were followed by irradiation, further analysis of the Survival Fractions (SF)—data not shown—and the Radiation Enhancement Ratio (RER) were performed as described in Subiel et al.^62^.

For annexin/PI analysis, both floating and adherent cells were harvested and analysed with the FITC Annexin V Apoptosis Detection Kit I (BD Bioscience), according to the manufacturer’s instructions. Cells were analysed utilizing the FACS flow LSR II (BD Bioscience).

### Cell cycle analysis

Both floating and adherent cells were harvested at 72 h from the treatment (as described previously). After being washed in a solution of FBS in PBS (1:100), cells were fixed by adding ice cold 100% Ethanol to the solution of FBS-PBS (4:1). Afterwards, cells were stained by replacing the solution containing ethanol with another made up of PBS/FBS and implemented with PI (1:100, BD Bioscience) and RNaseA (1:400, Life Technologies). Cells were then analysed through FACS flow LSR II (BD Bioscience).

### Analysis of Synergism/Antagonism in combination treatments

The nature of the interaction between ONC201 and X-rays was determined using the CompuSyn software (ComboSyn, Inc. and Ref.^63^). For the purpose, the viability of cells after 72 h from the treatments with ONC201 and radiation (alone and as sequential treatment) were inserted in the matrix of the Software to generate the algorithm.

### RNA-Seq analysis

Cells were harvested at the time-points indicated in Fig. 3a. The RNA was extracted from the cells and collected utilizing the column-based method through the RNeasy MinElute Clean-up Kit (QIAGEN, Hilden, Germany), at a concentration of 25 ng/µL in 20 µL, according to the manufacturer’s instructions. The RNA library preparations were performed with the KAPA RNA HyperPrep Kit (KAPA Biosystems, Roche Holding AG) according to the manufacturer’s instructions. Sequencing was performed on Illumina Next Seq 500 (High out output (150)) at 75 P.E at 25 M reads/sample. This was performed by the Genomic Core Technology Unit, CCRCB, Queen’s University Belfast. Fastq files were generated using bcl2fastq version 2.19 using the default thresholds. Reads were aligned to version GRCh37/hg19 of the human reference genome using STAR (version 2.4.2a). Raw counts of reads mapped to genes were calculated using HT‐Seq (http://www.huber.embl.de/users/anders/HTSeq/doc/overview.html) and used as input for differential expression analysis using DESeq2^64,65^. Principal components analysis was employed to visualise the overall effect of experimental covariates and batch effects within the data. Read counts per genes files were generated and principal component analysis (PCA) performed (RStudio, Version 1.2.5033, release name "Orange Blossom" (RStudio Team (2020). RStudio: Integrated Development for R. RStudio, PBC, Boston, MA URL http://www.rstudio.com/). Gene sets were filtered to contain genes upregulated with radiation (ratio > 1.5) and downregulated with ONC201 (ratio < 0.5). Pathway analysis were performed on this gene list using Reactome (Reactome.org, ELIXIR) and Enrichr^66,67^. The data shown in the heatmaps are represented as log2 of counts per gene. The data have been submitted to NCBI/GEO (GSE136975).

### Clonogenic assays

Clonogenic assays were performed as previously reported^68^.

### *FOCI* detection

2 × 10^4^ cells were seeded on 13 mm sterilized coverslips and treated as described previously. Cells were fixed at each time point (1 h, 24 h and 72 h form each treatment). DNA damage was detected using the immunofluorescence assay as previously described^18^. Briefly, cells were fixed in cold 50:50 solution of acetone and methanol and therefore permeabilized in 0.5% Triton X-100 (Merks Chemicals, Darmstadt, Germany) in PBS for 20 min and then incubated for 60 min in blocking buffer (0.1% Triton X-100, 5% FBS in PBS). Anti-53BP1 rabbit monoclonal primary antibody (Novus Biological, Centennial, USA), diluted 1:5000 in blocking buffer was added to the cells. Samples were incubated at room temperature for 1hour. Secondary antibody (anti-rabbit Alexa Fluor 568, Life Technologies, Carlsbad, USA) diluted 1:2000 in blocking buffer was added and incubated for 1 h. Cells on cover slips were briefly dried before mounted on slides with Prolong Gold Antifade with DAPI (Thermo Fisher, Waltham, USA). The number of 53Bp1 positive *foci* per cell were counted and imaged with the Nikon Eclipse Ti fluorescent microscope (Nikon Instruments Inc., Melville, NY, USA) in the entire nucleus. Experiments were repeated three times and at least 50 cells per repeat, treatment and time point were analysed.

### Data analysis

All data are shown as mean ± S.D. Tests of significance were performed by one-way ANOVA test, using Sigma Plot software (Systat Software Inc, Evanston, IL, USA). To analyse the statistical significance of the number of *foci* detected as previously described, data were run through the ANOVA test on Ranks (for not normalised distributions) followed by the many-to-one comparisons (Dunnet’s Method). Also, in this case, Sigma Plot software was utilised. Significant changes had p-values ≤ 0.05 (*p ≤ 0.05; **p < 0.01; ***p < 0.001).

## Supplementary Information

Supplementary Information.
