# Supplementary material for: Modulating the unfolded protein response with ONC201 to impact on radiation response in prostate cancer cells
Source: Sci Rep. 2021 Feb 19;11:4252. doi: 10.1038/s41598-021-83215-y (PMC7896060; doi:10.1038/s41598-021-83215-y)
Supplement: Supplementary file 1 — Supplementary Information. [file 41598_2021_83215_MOESM1_ESM.pdf]

Modulating the unfolded protein response with ONC201 to impact on radiation response in prostate cancer cells

Francesca Amoroso[1,7*], Kimberley Glass[1], Reema Singh[7], Francisco Liberal[1,2], Rebecca E. Steele[1,3], Sarah Maguire[1], Rohinton Tarapore[4], Joshua E. Allen[4], Sandra Van Schaeybroeck[1], Karl T. Butterworth[1], Kevin Prise[1], Joe M. O'Sullivan[1,5], Suneil Jain[1,5], David J. Waugh[6] and Ian G. Mills[1,7*].

[1] Centre for Cancer Research and Cell Biology, Queen's University Belfast, United Kingdom, BT9 7AE

[2] Faculdade de Ciências e Tecnologia, Universidade Nova de Lisboa, Portugal, 2825-516

[3] Breast Cancer Now Toby Robins Research Centre, The Institute of Cancer Research, London, United Kingdom, SW3 6JB

[4] Research & Development, Oncoceutics Inc., Philadelphia, PA 19104, USA

[5] The Northern Ireland Cancer Centre, Belfast City Hospital, United Kingdom, BT9 7AB

[6] Queensland University of Technology, Brisbane City QLD 4000, Australia

[7] Nuffield Department of Surgical Sciences, University of Oxford, John Radcliffe Hospital United Kingdom, OX3 9DU

Supplementary Information

Supplementary - Material and Methods

Cell lines and reagents. Prostate adenocarcinoma PC3 cell line and colorectal cancer HT-29 cell lines were obtained from the American Type Culture Collection (Authentication by short tandem repeat profiling/karyotyping/isoenzyme analysis). PC3 cells were maintained in RPMI-1640 (GIBCO-Life Technologies, Thermo Scientific, UK) implemented with 10% FBS (GIBCO-Life Technologies), 2 mM L-Glutamine, 100 U/ml penicillin and 100 mg/ml streptomycin (GIBCO-Life Technologies). HT-29 cells were maintained in DMEM (GIBCO-Life Technologies, Thermo Scientific, UK) implemented with 10% FBS (GIBCO-Life Technologies), 2 mM L-Glutamine, 100 U/ml penicillin and 100 mg/ml streptomycin (GIBCO-Life Technologies). Cells were maintained at 37°C in a 5% CO_2-humidified atmosphere. All experiments were performed in complete medium. Cells were plated overnight at a known density. The medium was replaced and implemented with several different treatments (either drugs or radiation) up to the next 72 hours. When cells were irradiated medium was replaced immediately prior to each irradiation dose using an XRad 225 cabinet source at dose rate 0.59 Gy/min, (Precision X-ray Inc, North Branford, CT, USA). Unexposed controls were prepared and treated as sham exposures (mock). ONC201 has been kindly provided by Oncoceutics (Philadelphia, PA, USA). ONC201 was dissolved as a stock solution of 20 mM in DMSO and used for experiments at a final concentration of 5, 10 and 15 µM in complete medium. BI2536 and Dinaciclib were purchased from Selleckchem (Munich, Germany). BI2536 was prepared as a stock solution of 10 mM in DMSO and used for experiments at a final concentration of 100 nM in complete medium [1]. Dinaciclib was prepared as a stock solution of 199.25 mM in DMSO and used for experiments at a final concentration of 0.1 nM in complete medium. The Vehicle was in all cases DMSO 0.1% in

complete medium. AT7519 (Sigma Aldrich) was used as positive control in the characterization of the mitochondrially depleted PC3 cells at a final concentration of 250 nM [2].

Depletion of mitochondrial DNA in PC3 cells. Mitochondrially depleted/Rho°cells PC3 cells were generated by continuous culturing of cells for 7 days with a nucleoside analogue, 2',3' dideoxycytidine (ddc, 10 µM) as mentioned in [3]. In brief, cells were cultured in high glucose (4.5 g/l glucose) Dulbecco's Modified Essential Medium supplemented with 10 % fetal bovine serum, 1% penicillin/streptomycin, 20 mM pyruvate, 2 mM glutamine and 50 µM Uridine (all Sigma Aldrich).

Western Blots. Cells were treated with ONC201 alone for 24 hours (Washouts) and up to 72 hours, radiation alone (single doses of 2Gy, 4Gy and 8Gy) and as a sequential treatment with ONC201 for 24 hours followed by radiation in single dose (a schematic view of the treatments is in Figure 3, a). Cells were detached trough trypsinization (5 minutes at 27˚C, Sigma Aldrich) at 24 hours and 72 hours after each treatment and lysed with Urea buffer (Urea 9 M, Tris Base 1 M, PMSF 10 mM, beta-mercaptoethanol 1%, (all Sigma Aldrich). Proteins were separated on 4-12% NuPage precast gels (Life Technologies) and blotted on nitrocellulose paper (Life Technologies). Membranes were cut at the specific Molecular Weights of 100, 100-70, 70-35 KDa (utilizing the Marker -Thermo Scientific Page Ruler Prestained Protein Ledder- as a reference) to accommodate all the proteins of interest. The portioned membranes were then incubated overnight with antibodies anti- IRE1a (14C10), BiP (C50B12), PERK (D11A8), CHOP (L63F7), PLK1 (208G4), PhAkt(Ser473), Akt (all Cell Signaling, Danvers, MA, USA), ATF4 (EPR18111) (Abcam, Cambridge, UK), ATF6 (70B1413.1) (Novus, Manchester, UK), DRD2 (Millipore, Massachusetts, USA), PARP (46D11) and Caspase 3 (all detecting both full length and cleaved forms, Cell Signaling) at a dilution of 1:1000 in a solution of 3% not fat milk-PBS. Antibody anti-XBP1 spliced (143F) (Biolegend, San Diego, CA, USA), was used at a dilution of 1:400 in 3% not fat milk-PBS.

The anti-tubulin Ab (Cell Signaling) was used as housekeeping control at a dilution of 1:1000 as described above. The secondary anti-rabbit and anti-mouse horseradish peroxidase-conjugated Abs (Cell Signaling) were used at a dilution of 1:3000 in PBS-0.25% Tween (Sigma Aldrich). Where necessary, the portioned membranes were stripped using Restore™ Western Blot Stripping Buffer (Thermo Scientific) for 7 minutes before a second round of incubation with different antibodies took place. Protein bands were revealed with the Western Lightning Ultra kit (PerkinElmer, Waltham, MA, USA) using the GBox Imager by Syngene (Cambridge, UK). Densitometic analysis of the blots (n=3) were performed utilizing ImageJ software (Public Domain, BSD-2).

qRT-PCR and primers. To perform the RNA extraction, cells were treated and harvested at each time point trough trypsinization (as described above) and processed with Trizol (Life Technologies) according to the manufacturer's instructions. qRT-PCR analysis was performed using the LightCycler® 480 probes master mix and LightCycler®480II (both Roche, Basel, Switzerland). RPLPO was used as a housekeeping gene. The primers (all from Eurofins Genomics UK) used in this work have the following sequences:

targeted gene (protein)	Forward sequence (5'-3')	Reverse Sequence (5'-3')
RRM2	tggacctctccaaggacatt	ggctaaatcgctccacca
ZBTB20	atgggtgcacacaggaaaat	cttctcatgggcctgtatgtt
MKI67 (Ki67)	gagagtaacgcggagtgtca	tcactgtccctatgacttctgg
TYMS	cccagtttatggcttccagt	gcagttggtcaactccctgt
PLK1	aagatctggaggtgaaaataggg	aggagtcccacacagggtct
CDK1	tggatctgaagaaatacttggattcta	tctggagatctgtaccagagtgtt
CDK2	cctcctgggctgcaaata	cagaatctccagggaataggg

RPLPO	atcaacgggtacaaacgagtc	cagatggatcagccaagaagg

References

1. Wissing MD, M.J., Kortenhorst MS, Kaelber NS, Gonzalez M, Kim E, Hammers H, van Diest PJ, Carducci MA, Kachhap SK. Targeting prostate cancer cell lines with polo-like kinase 1 inhibitors as a single agent and in combination with histone deacetylase inhibitors. *FASEB J.* **27**, 4279-93 (2013).
2. Itkonen HM, P.N., Walker S and Mills IG. CDK9 Inhibition Induces a Metabolic Switch that Renders Prostate Cancer Cells Dependent on Fatty Acid Oxidation. *Neoplasia* **21**, 713-720 (2019).
3. Ashley, N., Harris, D. & Poulton, J. Detection of mitochondrial DNA depletion in living human cells using PicoGreen staining. *Exp Cell Res* **303**, 432-46 (2005).
4. Oliveros, J.C. Venny. An interactive tool for comparing lists with Venn's diagrams. *http://bioinfogp.cnb.csic.es/tools/venny/index.html* (2007-2015).

Supplementary Figure Legends

Supplementary Figure 1. a-c) Characterization of the mitochondrially depleted cell line (mt depleted PC3, see Methods for details) (n=3). **a)** Evaluation of the relative mitochondrial DNA content in mt depleted PC3 cells through RT-PCR upon treatment with ddc (10 µM) for 8 days. **b-c)** Evaluation of different respiratory parameters in mt depleted PC3 cells upon treatment with ddc (10 µM) and AT7519 (250 nM), for 8 days (n=3). Seahorse analysis showing basal Oxygen Consumption Rate (OCR, b), basal respiration, maximal respiration, proton leak and ATP production (c) of mt depleted PC3 cells (see Methods for details). AT7519 was used as an experimental positive control as described in Itkonen *et al.* [2] . **d)** Mitochondrial stress test performed utilizing the Seahorse assay (see Methods for details) showing the bioenergetics parameters (OCR /pmol/min) of the PC3-WT and mt depleted PC3 cells upon treatment with ONC201 (10 µM) and Vehicle for 24 hours. All cell lines were cultured in respective media (see Methods for details). **e)** Representation of the Basal OCR and the extracellular acidification rate (ECAR) levels upon treatment of PC3-WT cells and PC3-mitochondrially depleted (mt depleted PC3) cells with Vehicle and ONC201 (10 µM) for 24 hours. Representative traces are shown in Supplementary figure 2, d. **f)** The mitochondrial stress test and the OCR measurements were performed as described in d) to evaluate the bioenergetics parameters of the PC3-WT and mt depleted PC3 upon treatment with ONC201 (10 µM) for 24 hours. All cell lines were cultured in media with high glucose (see Methods for details). **g)** Measurement of the OCR in PC3-WT cells and mt depleted PC3 cells cultured in media with high glucose (see Methods for details) and treated with Vehicle or ONC201 (10 µM) for 24 hours. The measurements were taken after the addition of oligomycin (see Methods for details) and normalized by cell numbers for each experiment by utilising the CyQuant cell count assay. **h)** Seahorse analysis of basal OCR and the

extracellular acidification rate (ECAR) levels in PC3-WT cells and mt depleted PC3 cells (as described in (e). All cell lines were cultured in media with high glucose (see Methods for details). A one-way ANOVA test was performed comparing treatments to controls (either Vehicle or mock radiated) on n=3 experiments, * p≤0.05; **p<0.01; *** p<0.001 (±SD).

Supplementary Figure 2. a) Western Blot analysis showing the expression of all the components of the UPR in PC3 cells primed to radiation (Xrad, 2-8 Gy) with ONC201 (5, 10 and 15 µM) for 24 hours (representative of n=3). Samples were harvested at 24 hours from the last radiation (left panel). Samples treated with ONC201 for 24 hours only and harvested at 72 hours from the washouts (ONC201 W.O.) are also shown (middle panel). Samples harvested at 72 hours from the last radiation are shown in the right panel. The membranes were cut before the incubation with the primary antibody (see Material and Methods for specifications) and either detected alone or together. All the membranes were exposed for 3 minutes, with the exception of ATF4, ATF6 (exposed for 6 minutes), PERK and Tubulin (exposed for 2 minutes). **b)** Densitometric analysis of Western Blots performed on samples treated as described in a) are shown and are relative to the samples harvested 24 hours post-radiation or as washouts (W. O.) (n=3 ±SD). **c)** Densitometric analysis of Western Blots performed on samples treated as described in a) are shown and are relative to the samples harvested 72 hours post-radiation (also shown in Fig. 2, e) (n=3 ±SD). One-way ANOVA test was performed comparing treatments to controls (either Vehicle or mock radiated) on n=3 experiments, * p≤0.05; **p<0.01; *** p<0.001 (±SD).

Supplementary Figure 3. a) PCA plots of RNAseq analysis performed on PC3 cells primed to radiation (Xrad) with ONC201 for 24 hours showed two subgroups of differentially expressed gens (samples harvested at 24 hours from radiation –red dots- and at 72 hours – blue dots) (RStudio software, Version 1.2.5033, release name "Orange Blossom" (RStudio Team (2020). RStudio: Integrated Development for R. RStudio, PBC, Boston, MA URL http://www.rstudio.com/). **b)**

Pathway analysis on the most differentially expressed genes (Ratio> 1.5 of radiated samples vs mock treated samples and Ratio< 0.5 of ONC201 treated samples vs Vehicle), shortlisted from the RNA-seq analysis, revealed that the most impacted pathway is cell cycle related (http://cpdb.molgen.mpg.de/ - p-value cut-off = 0.01 with minimum overlap with input list = 2). **c)** The Venn diagram graphically represents the intersection between the most differentially expressed genes shortlisted from the RNA-Seq analysis (Ratio> 1.5 of radiated samples vs mock treated samples and Ratio< 0.5 of ONC201 treated samples vs Vehicle) -and listed in table 1- in relation to their direct regulation of the most effected pathways (Venny 2.1.0 [4]).

Supplementary Figure 4. a) Total number of 53BPp1$^+$ *foci* per cell at 72 hours post irradiation. Samples were analysed at the time points schematically represented in Figure 3, a (n=3). The data have been run through ANOVA test on Ranks and further analysed with Dunnett's Method. * $p \leq 0.05$; **$p<0.01$; *** $p<0.001$ (±SD). **b)** Immunofluorescence analysis of 53BPp1$^+$ *foci* in PC3 cells treated as schematically represented in Figure 3, a. Single channels of the *foci* determination at 1 and 24 hours from radiation are shown - 53BPp1 (red) and DAPI (blue).

Supplementary Figure 5. a) Western Blot analysis showing the reduced expression of PLK1, one of the major check points of the S/G_2M transition through the cell cycle, in PC3 cells treated with ONC201 (5, 10 and 15µM). Samples were treated as shown in Figure 2, (a) and harvested at 72 h from the last radiation (representative of n=3). The membranes were cut before the incubation with the primary antibody (see Material and Methods for specifications) and either detected alone or together. The exposure time was of 5 minutes for PLK1 and 2 minutes for the Tubulin. Densitometric analysis of n=3 WB are shown, and the average is expressed as a number embedded within the figure (the SD are within the 5% acceptance of statistical significance). **b)** Cell cycle analysis at 72 hours of PC3 cells primed for 24 hours to radiation (2-8Gy) with BI2536 (100 nM). The top panels are representative of n=3 experiments and showed the predicted effect that treating PC3 cells with

BI2536 (100 nM) alone for 72 hours has on the cell cycle progression (Quality control). Priming PC3 cells with BI2536 (100 nM) for 24 hours before radiation increases the percentage of population exiting the G0/G1 phase (ochre) and transitioning into the S and G2/M phases (grey and yellow respectively) of the cell cycle (bottom panel, n=3) at 72 hours. **c)** Priming PC3 cells with BI2536 (100 nM) for 24 hours before radiation increases cell death through Necrosis (green) and Apoptosis (red and blue) at 72 hours (n=3). **d-e)** Cell counts of PC3 cells surviving hypo-fractionated doses of radiation (d, 5 x 2Gy – e, 10 x 2Gy respectively) and afterwards treated with (5 µM and 10 µM) up to 72 hours. One-way ANOVA was performed on all n=3 comparing treatments to controls (either Vehicle or mock radiated), * $p \leq 0.05$; **$p<0.01$; *** $p<0.001$ (±SD).

Supplementary Figure 6. a) Cell cycle analysis of PC3 treated with subtoxic concentration of Dinaciclib (0.1 nM), CDKs inhibitors, for 24 hours prior to radiation (2, 4 and 8Gy). Samples were harvested and analysed at 72 hours. **b)** Cell death analysis through AnnexinV/PI staining of PC3 treated with subtoxic concentration of Dinaciclib (0.1 nM), for 24 hours prior to radiation (2, 4 and 8Gy). Samples were harvested and analysed at 72 hours. One-way ANOVA was performed comparing treatments to controls (either Vehicle or mock radiated) on n=3 experiments, * $p \leq 0.05$; **$p<0.01$; *** $p<0.001$ (±SD).

Supplementary Figure 7. a) Original membranes utilised for the western blots shown in Figure 1, b (PC3 cells). The membranes were cut before the incubation with the primary antibody (see Material and Methods for specifications). **b)** Original membranes for western blots in Figure 1, e (PC3 cells). The membranes were striped between each incubation with a new primary antibody (see Material and Methods for specifications). **a-b)** Highlighted with a red box is the portion of the membrane utilized to generate the main figure. All the membranes were exposed for 5 minutes, with the exception of PARP (exposed for 7 minutes), Caspase 3 (exposed for 6 minutes) and GAPDH (exposed for 2 minutes).

Supplementary Figure 8. a) Original membranes utilized for the western blots shown in Figure 2, e (PC3 cells). The samples were treated with ONC201 or Radiation as monotherapy or as sequential treatment. The membranes were cut before the incubation with the primary antibody (see Material and Methods for specifications) and either detected alone or together. **b)** Original membranes for the western blots shown in Figure 6, a (HT-29 cells). The samples are treated with ONC201 for 24 hours. **a-b)** Highlighted with a red box is the portion of the membrane utilized to generate the main figure. All the membranes were exposed for 3 minutes, with the exception of ATF4, ATF6 (exposed for 6 minutes), PERK and Tubulin (exposed for 2 minutes).

Supplementary Figure 9. a) Original membranes utilized for the western blots shown in Supplementary Figure 2, a – 24 hours (PC3 cells). The samples were treated with ONC201 and radiation as monotherapy or as sequential treatment. The membranes were cut before the incubation with the primary antibody (see Material and Methods for specifications) and either detected alone or together. **b)** Original membranes utilized for the western blots shown in Supplementary Figure 2, a – Washouts (W.O.) (PC3 cells). The samples were treated with ONC201 as monotherapy for 24 hours, then harvested at 72 hours after ONC201 was removed. The membranes were cut before the incubation with the primary antibody (see Material and Methods for specifications) and either detected alone or together. **c)** Original membranes utilized for the Western Blots shown in Supplementary Figure 2, a – 72 hours (PC3 cells). The samples were treated with ONC201 and radiation as monotherapy or as sequential treatment. The membranes were cut before the incubation with the primary antibody (see Material and Methods for specifications) and either detected alone or together **a-c)** Highlighted with a red box is the portion of the membrane utilized for the main figure. All the membranes were exposed for 3 minutes with the exception of XBP1 (exposed for 7 minutes) and Tubulin (exposed for 2 minutes).

Supplementary Figure 10. a) Original membranes for the western blots shown in Supplementary Figure 5, a. The samples were treated with ONC201 and radiation as monotherapy or as sequential treatment. The membranes were cut before the incubation with the primary antibody (see Material and Methods for specifications) and either detected alone or together. Highlighted with a red box is the portion of the membrane utilized for the main figure. **b)** Western Blot showing the level of expression of DRD2 in PC3 cells and HT-29 cells. The samples are prepared as described in Material and Methods (representative of n=3). The membranes were cut just before the detection of the Tubulin (utilised as loading control). **c)** Western blot showing the level of expression of PhAkt(Ser473) and total Akt in PC3 cells treated with ONC201 (5-15 µM) for 24 hours (n=3). The GAPDH was utilised as loading control. Densitometric analysis are shown in the same panel. The level of expression of PhAkt(Ser473) was normalised on the level of expression of total Akt in each condition. One-way ANOVA was performed comparing treatments to controls on n=3 experiments showing that there was no significance (±SD). **a-c)** All the membranes were exposed for 3 minutes, with the exception of PLK1 (exposed for 5 minutes), and Tubulin (exposed for 2 minutes).

Supplementary Figure 11. a) Top panel. Densitometric analysis of western blots performed on samples treated with ONC201 as monotherapy for 24 hours are shown (representative western blots are shown in Figure 1, b) (n=3 ±SD) (PC3 cells). **Bottom panel.** Densitometric analysis of western blots performed on samples treated with ONC201 as monotherapy for 48 hours are shown (representative Western Blots are shown in Figure 1, e) (n=3 ±SD) (PC3 cells). **b)** Densitometric analysis of western blots performed on samples treated with ONC201 as monotherapy for 24 hours are shown (representative western blots are shown in Figure 6, a) (n=3 ±SD) (HT-29 cells). One-way ANOVA was performed comparing treatments to controls on n=3 experiments, **$p<0.01$; *** $p<0.001$ (±SD).

Supplementary Figure 12. a) Original membranes utilised for the Western Bots shown in **Supplementary Figure 10, b** (PC3 and HT-29 cells). The membranes were fully probed with the primary antibodies (see Material and Methods for specifications). **b)** Original membranes for the Western blots in **Supplementary Figure 10, c** (PC3 cells). The membranes were cropped before the incubation with the primary antibodies and striped between each incubation with a new primary antibody (see Material and Methods for specifications). **a-b)** Highlighted with a red box is the portion of the membrane utilized to generate the main figures. All the membranes were exposed for 5 minutes excepted Tubulin and GAPDH (exposed for 1 minute).

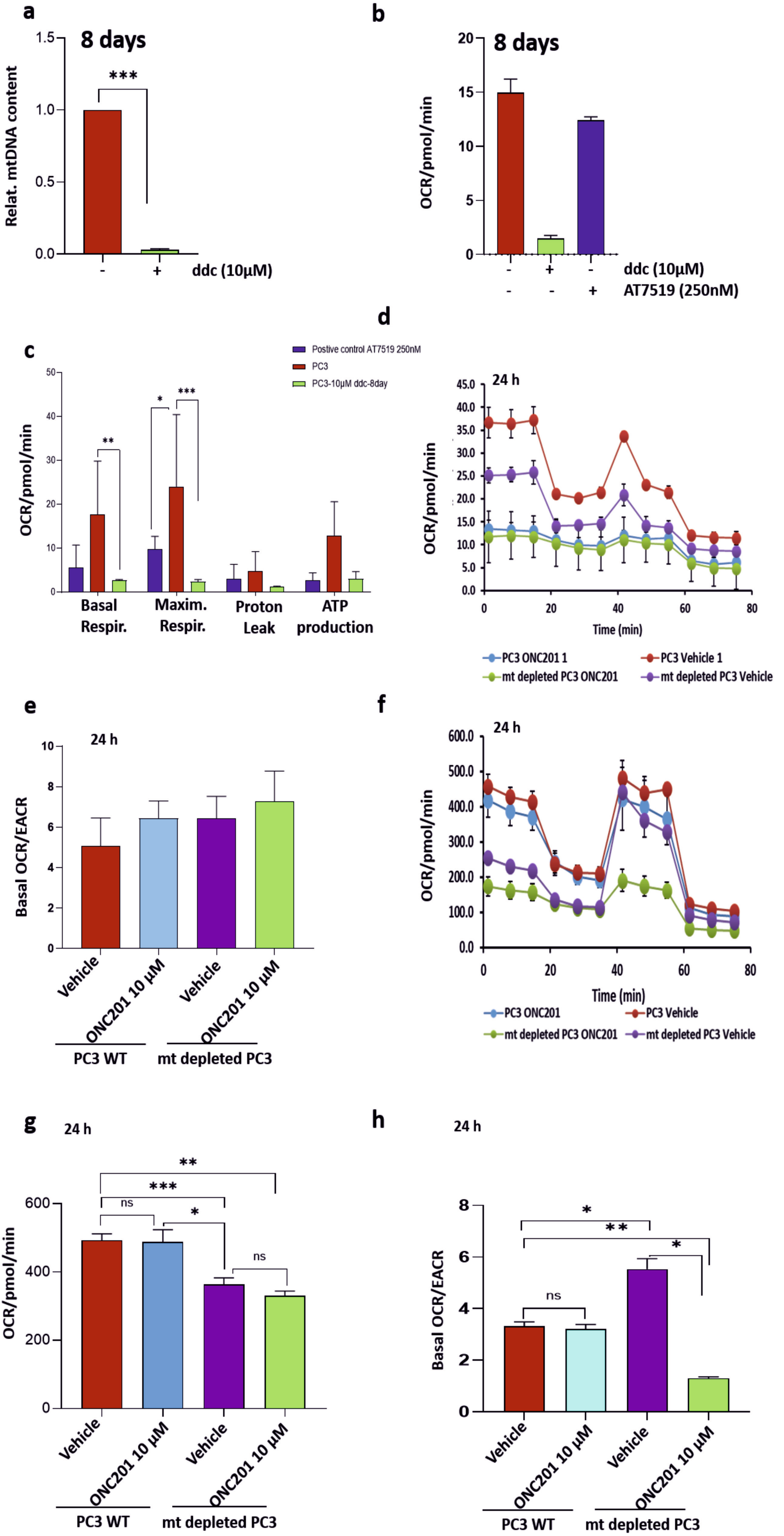

a
8 days
Relat. mtDNA content
***
ddc (10µM)
b
8 days
OCR/pmol/min
ddc (10µM)
AT7519 (250nM)
c
Postive control AT7519 250nM
PC3
PC3-10µM ddc-8day
OCR/pmol/min
Basal Respir.
Maxim. Respir.
Proton Leak
ATP production
d
24 h
OCR/pmol/min
Time (min)
PC3 ONC201 1
PC3 Vehicle 1
mt depleted PC3 ONC201
mt depleted PC3 Vehicle
e
24 h
Basal OCR/EACR
Vehicle
ONC201 10 µM
PC3 WT
mt depleted PC3
f
24 h
OCR/pmol/min
Time (min)
PC3 ONC201
PC3 Vehicle
mt depleted PC3 ONC201
mt depleted PC3 Vehicle
g
24 h
OCR/pmol/min
ns
Vehicle
ONC201 10 µM
PC3 WT
mt depleted PC3
h
24 h
Basal OCR/EACR
ns
Vehicle
ONC201 10 µM
PC3 WT
mt depleted PC3

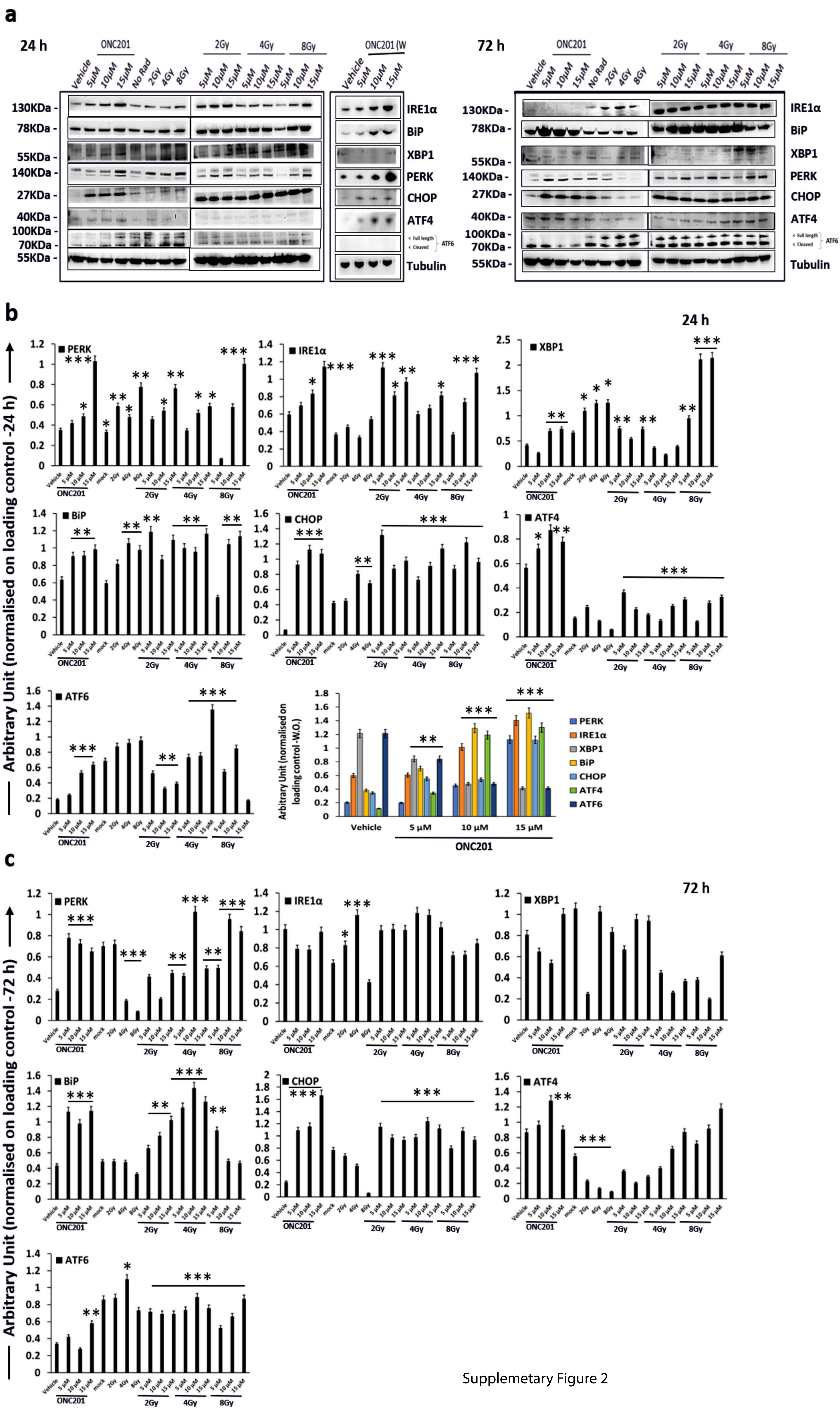

Supplemetary Figure 2

a

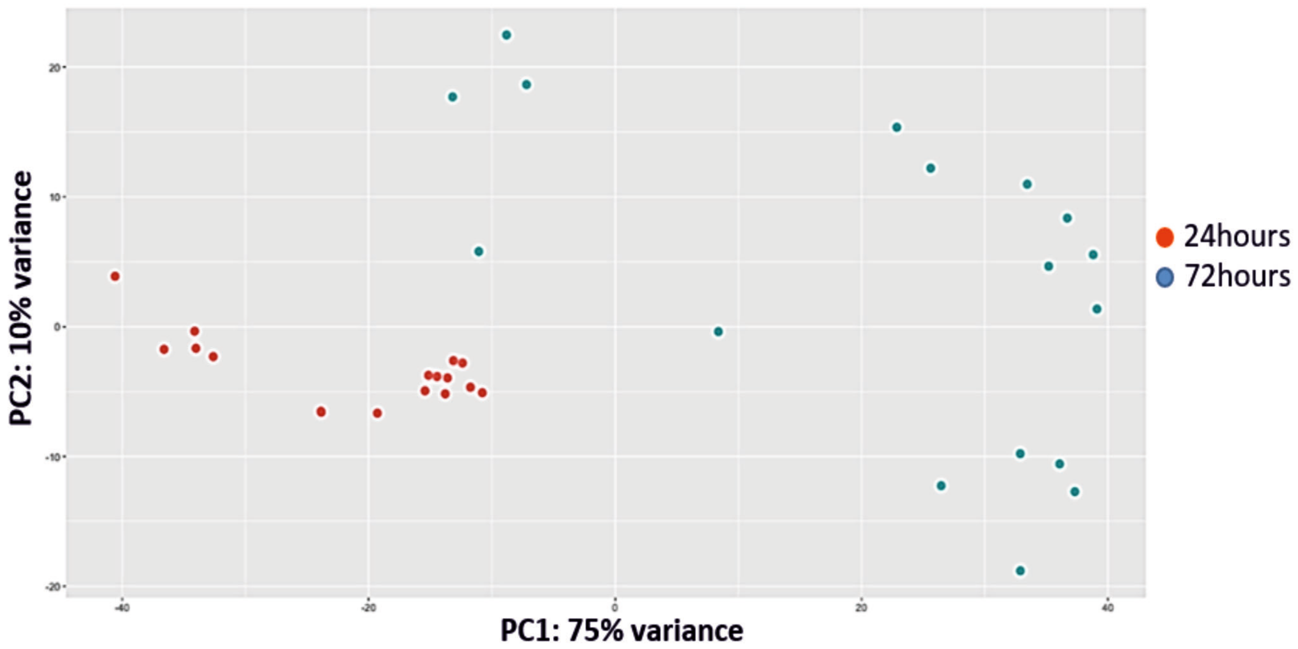

b

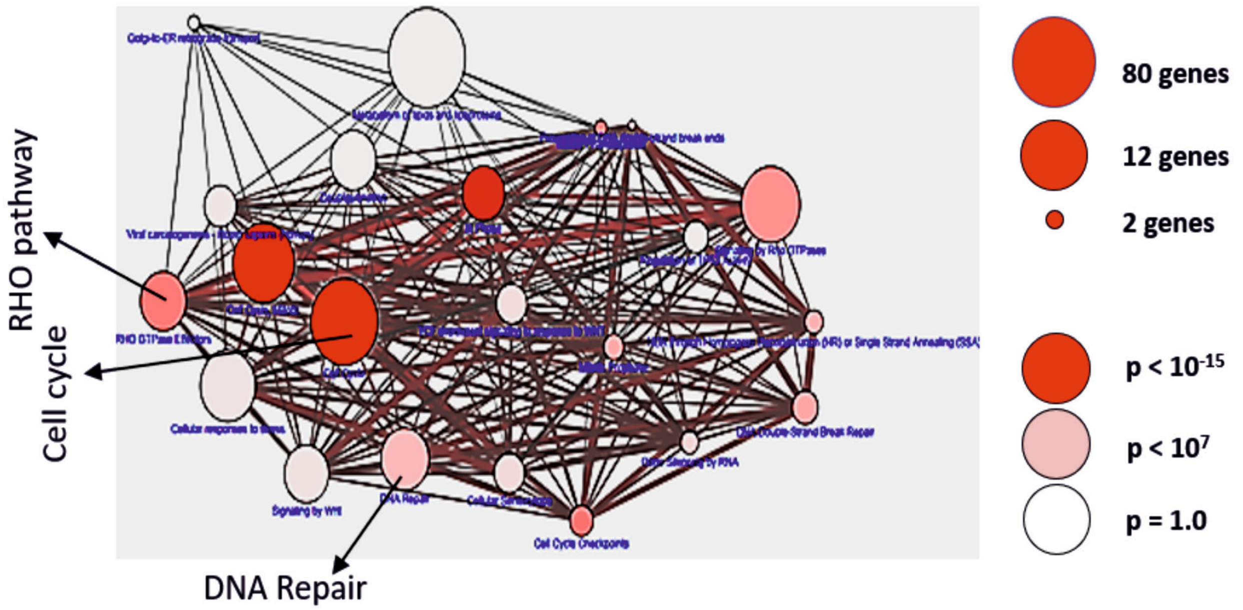

c

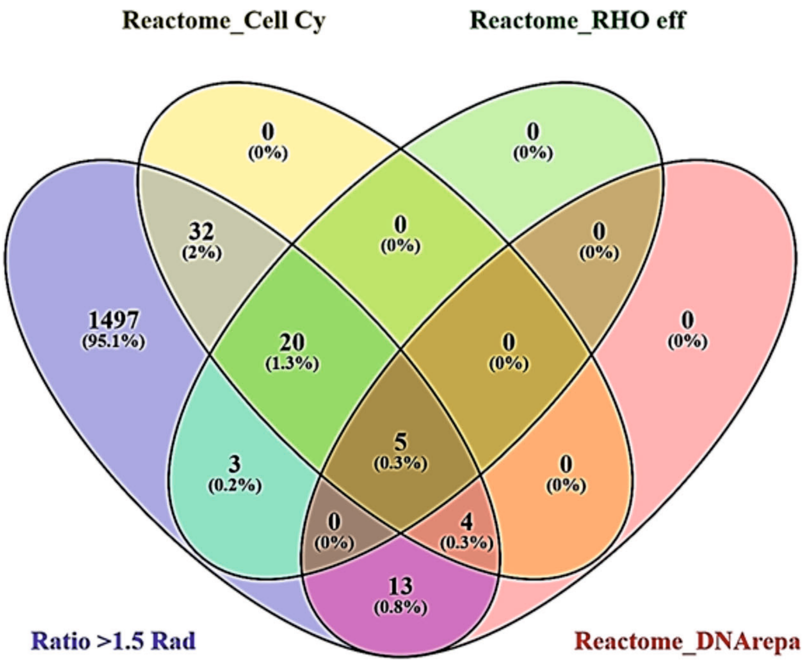

Supplementary Figure 3

a

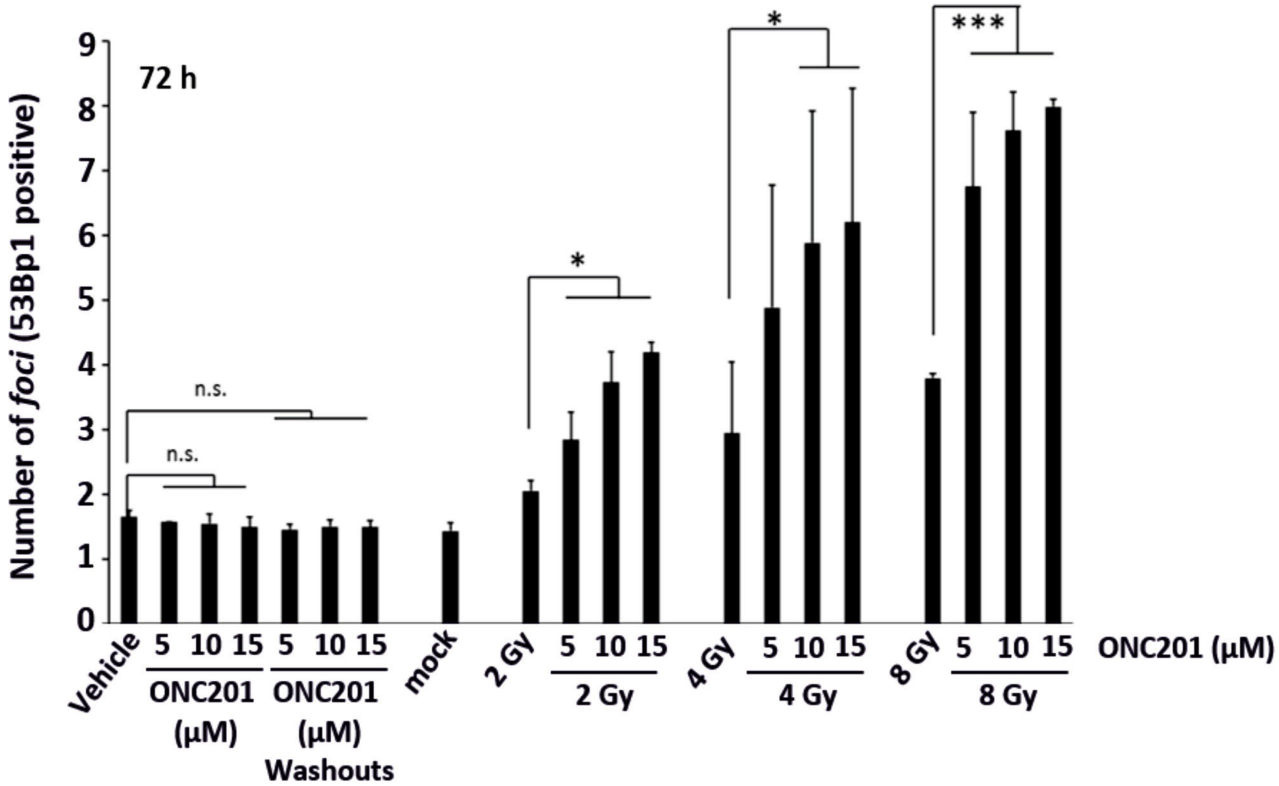

b

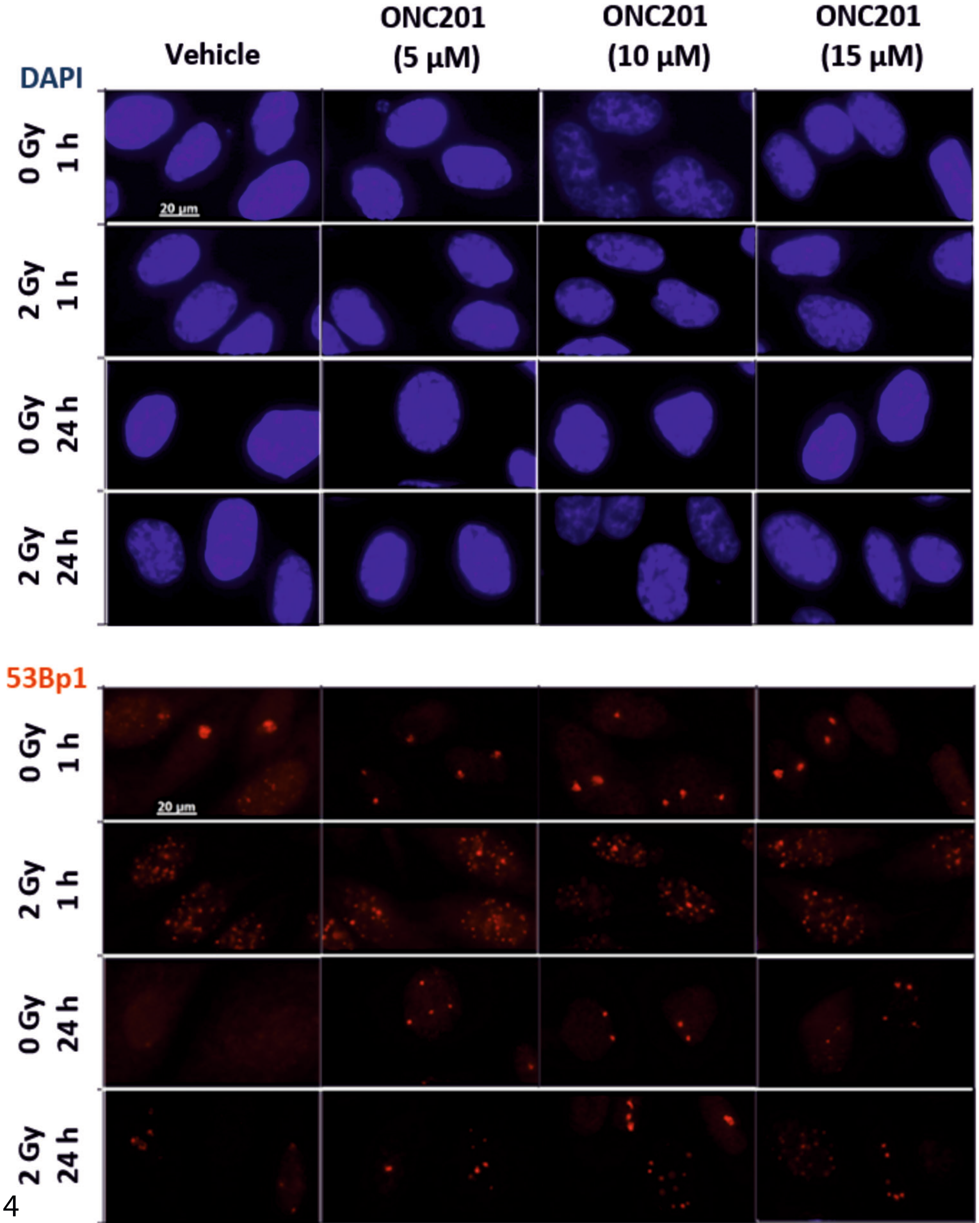

Supplementary Figure 4

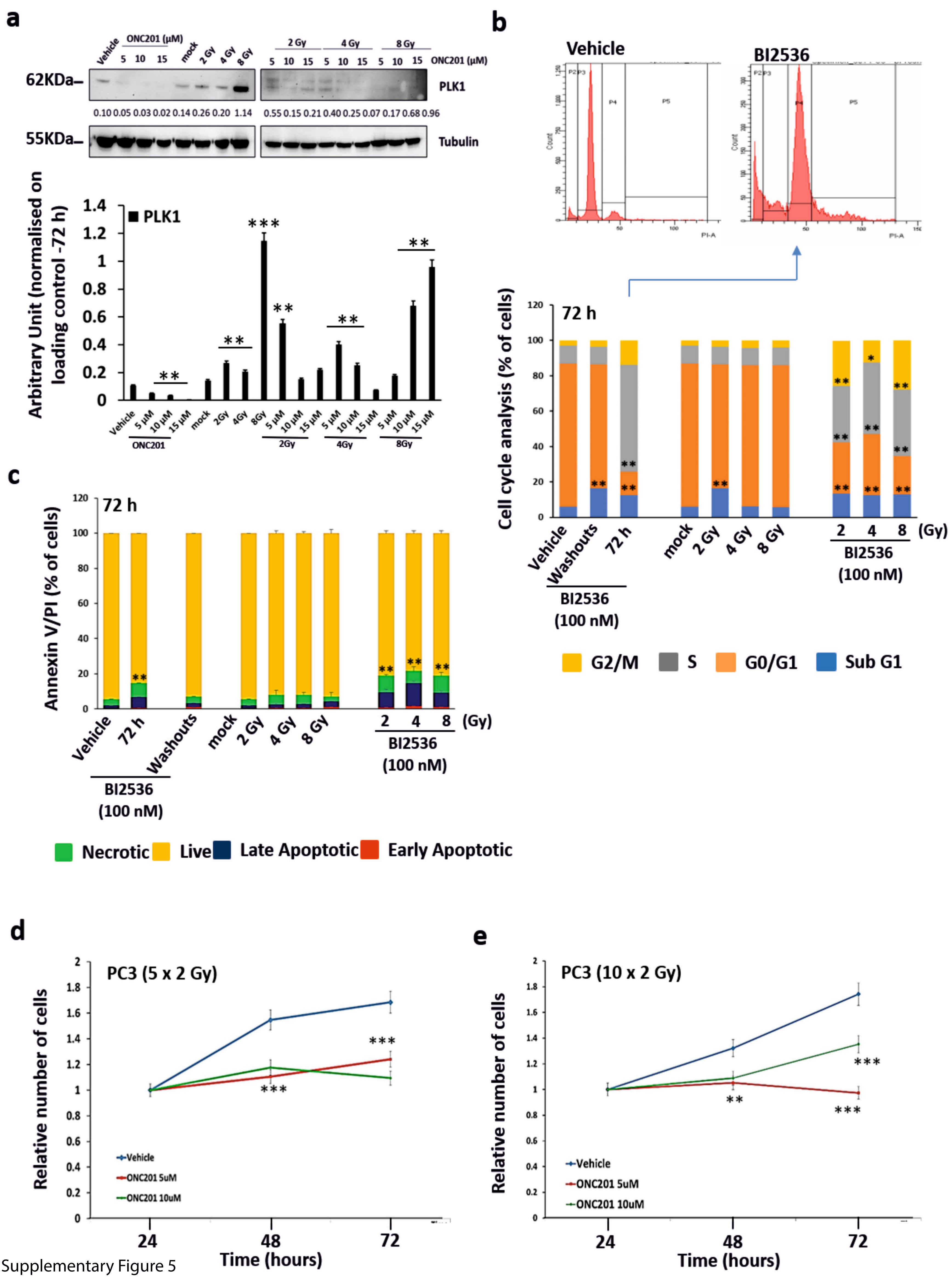

Supplementary Figure 5

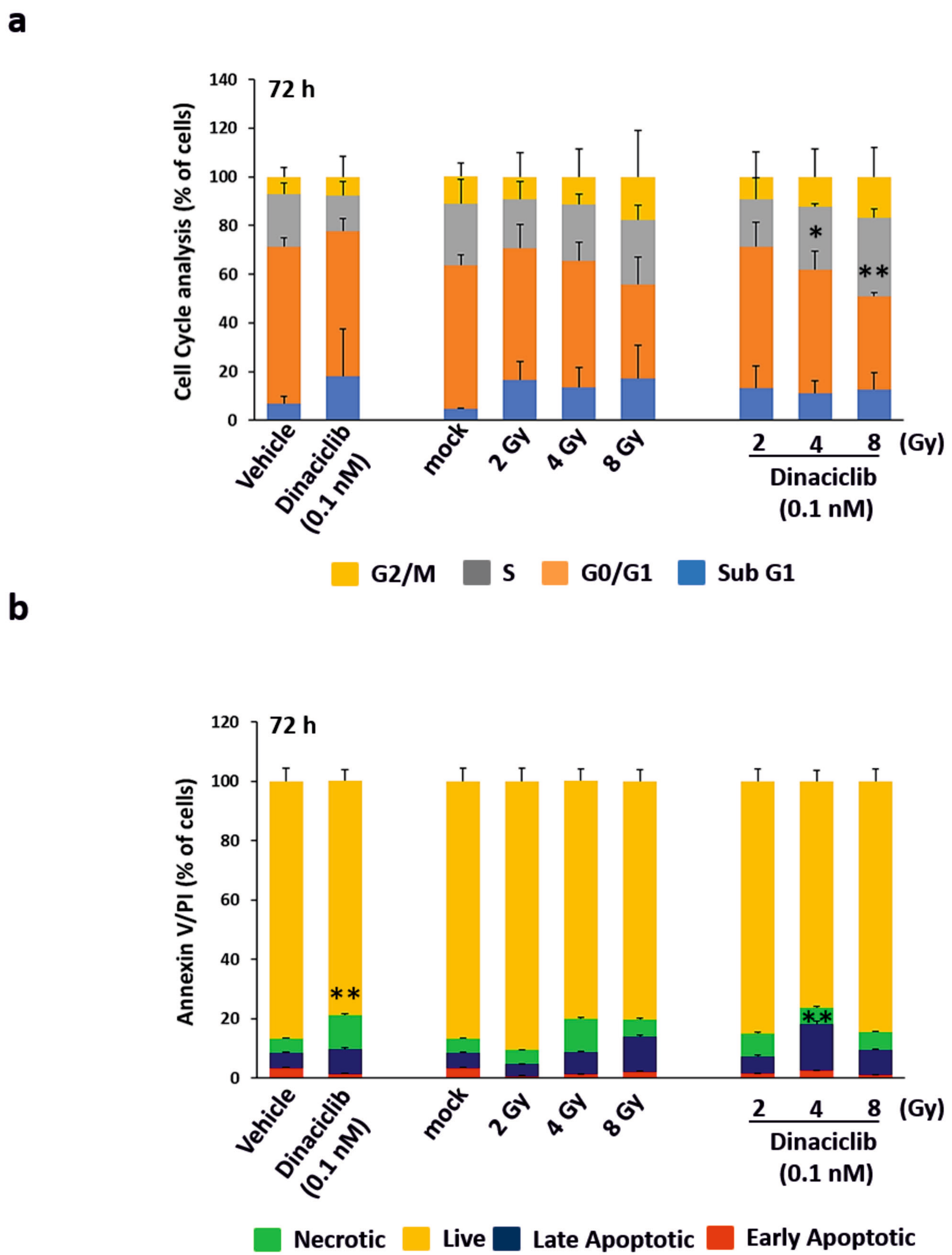

Supplementary Figure 6

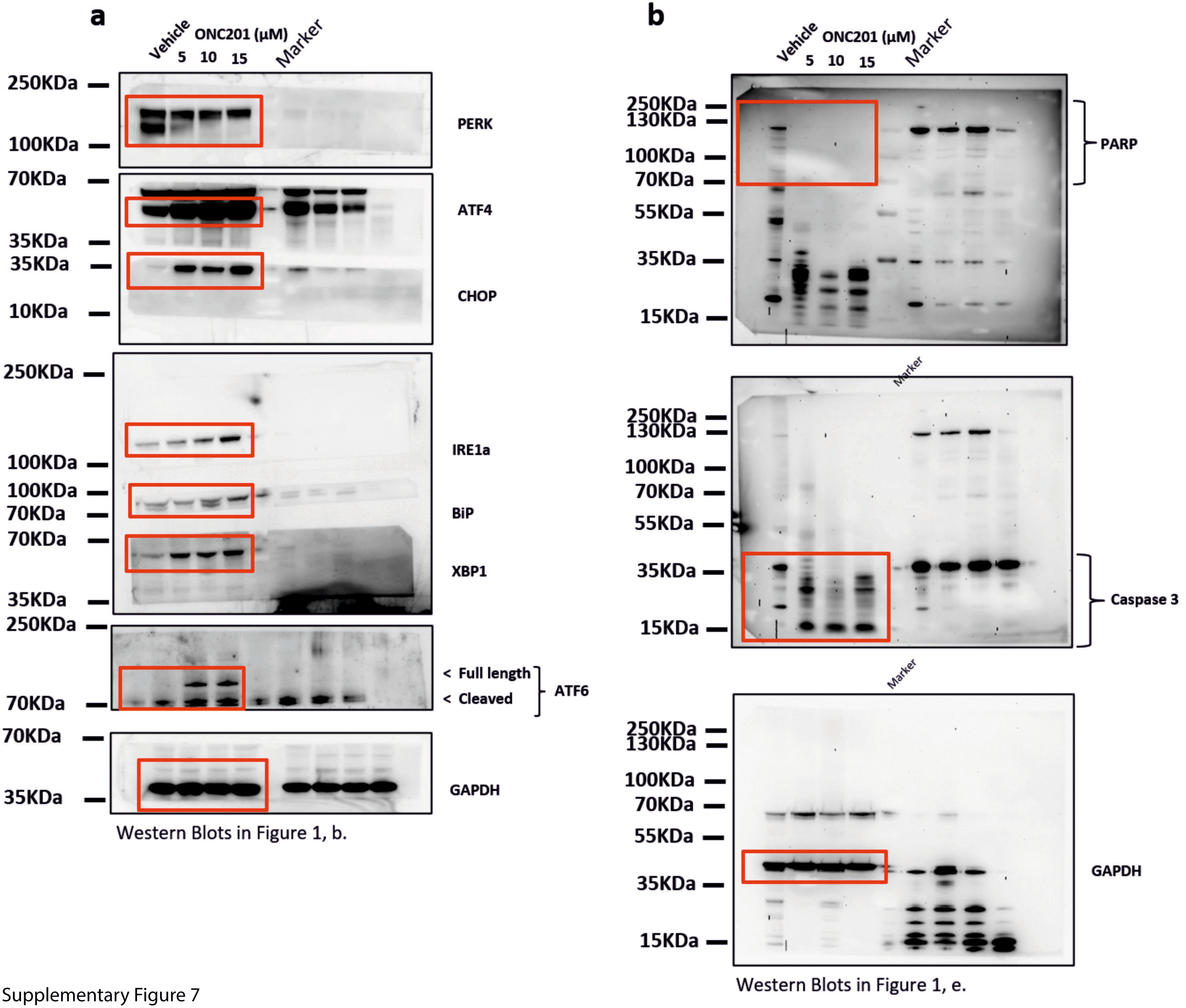

Supplementary Figure 7

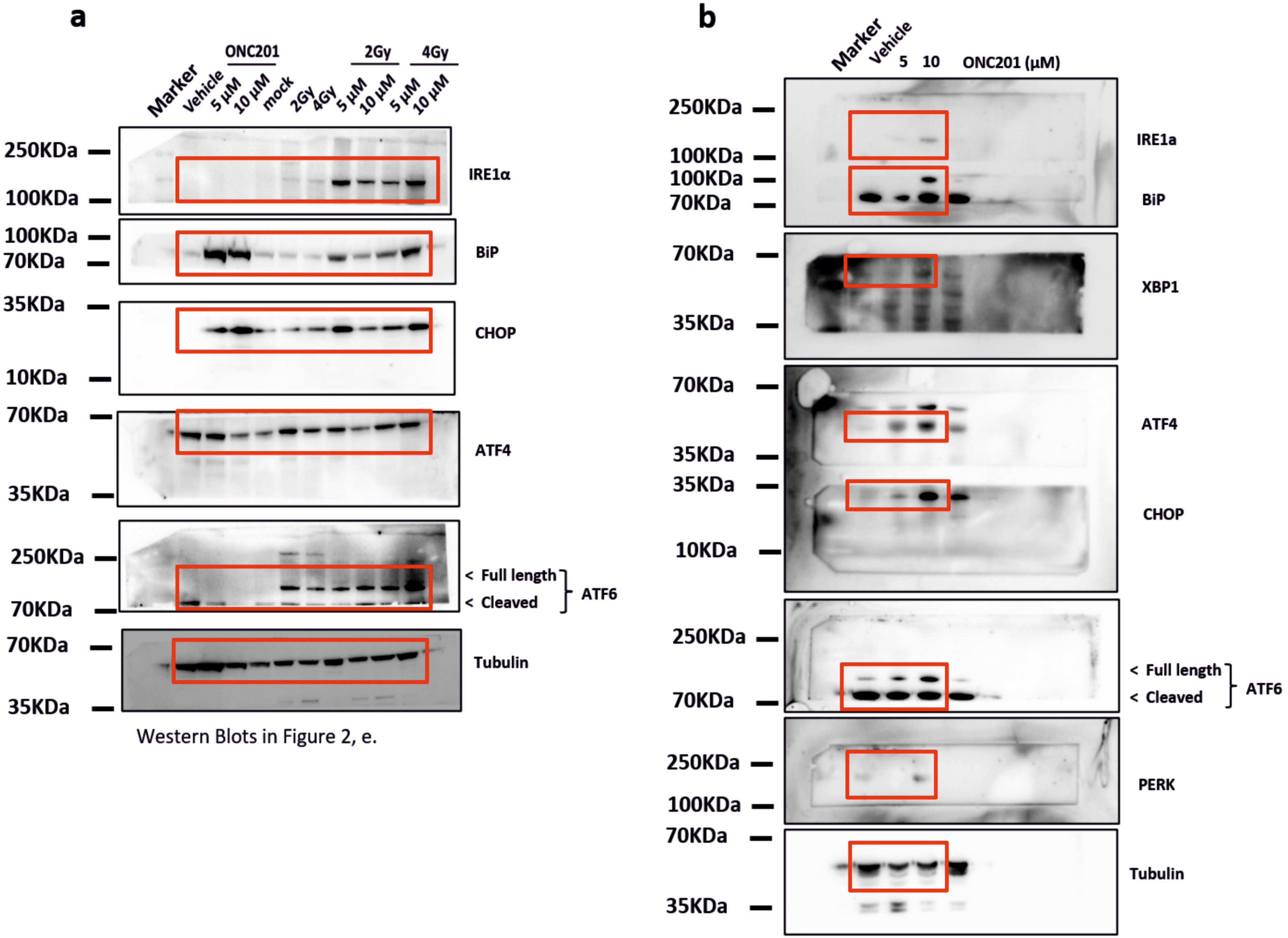

Western Blots in Figure 2, e.

Western Blots in Figure 6, a.

Supplementary Figure 8

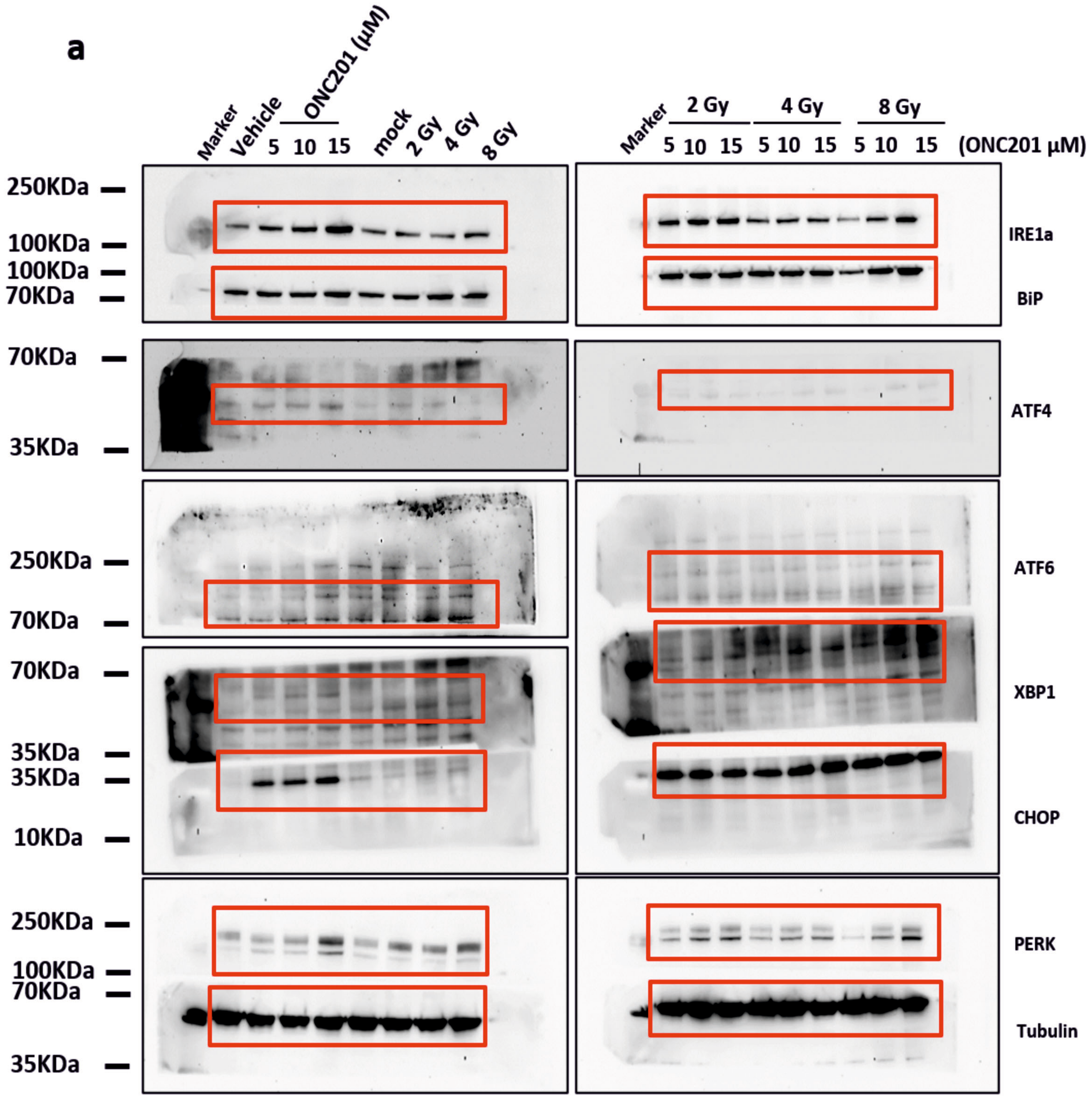

Western Blots in Supplementary Figure 2, a (24 h).

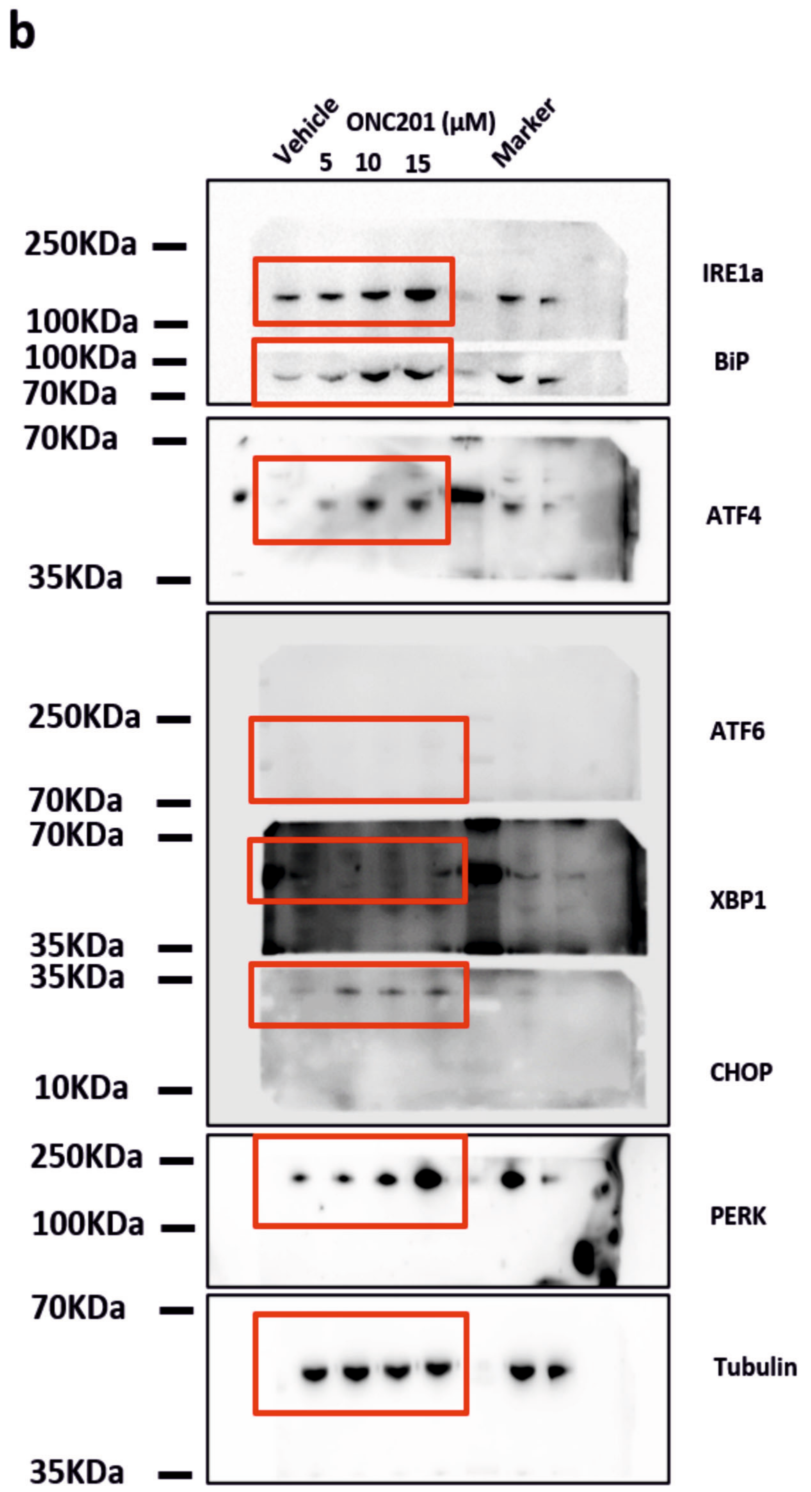

Western Blots in Supplementary Figure 2, a (ONC201 W.O.).

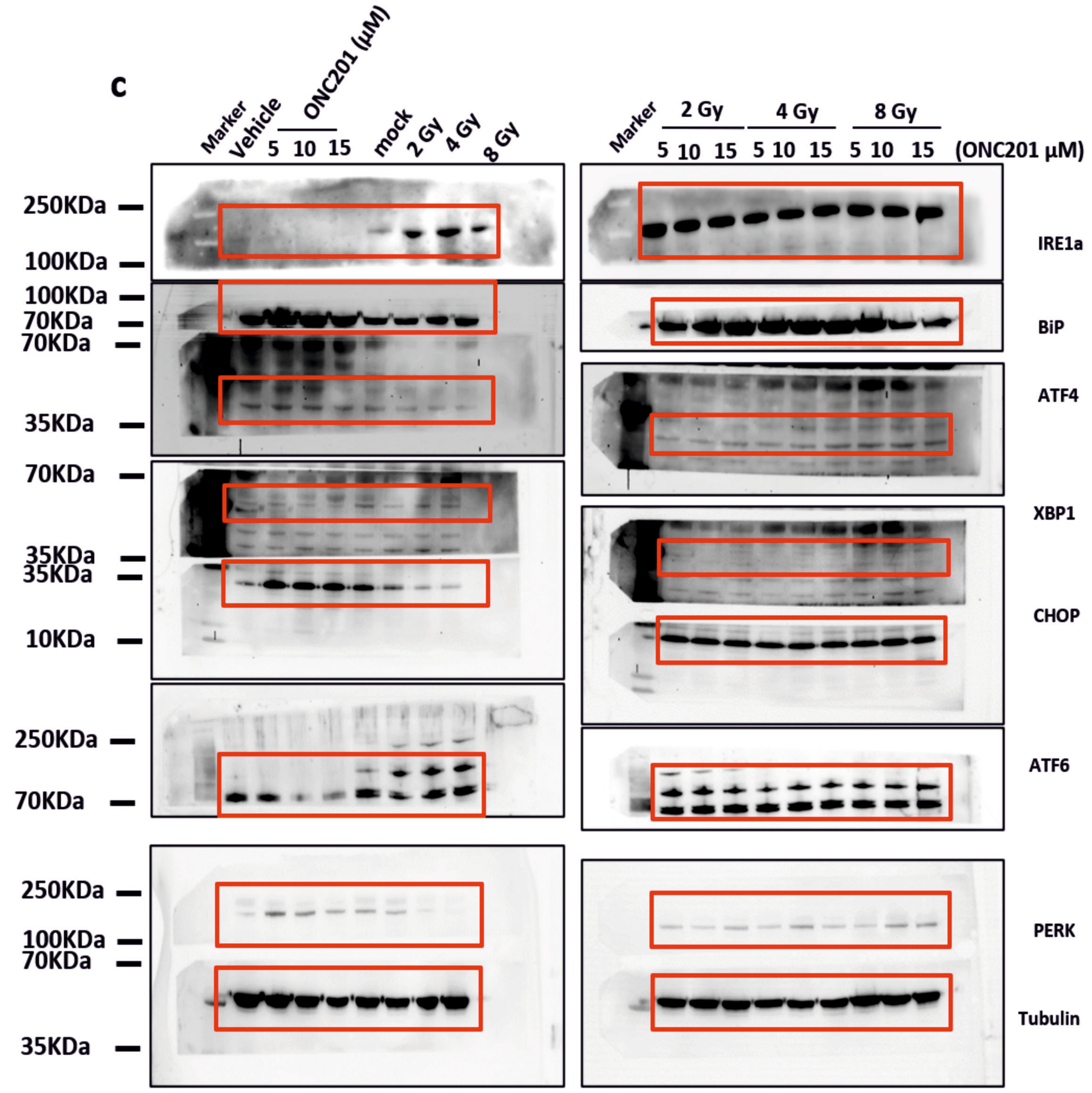

Western Blots in Supplementary Figure 2, a (72 h).

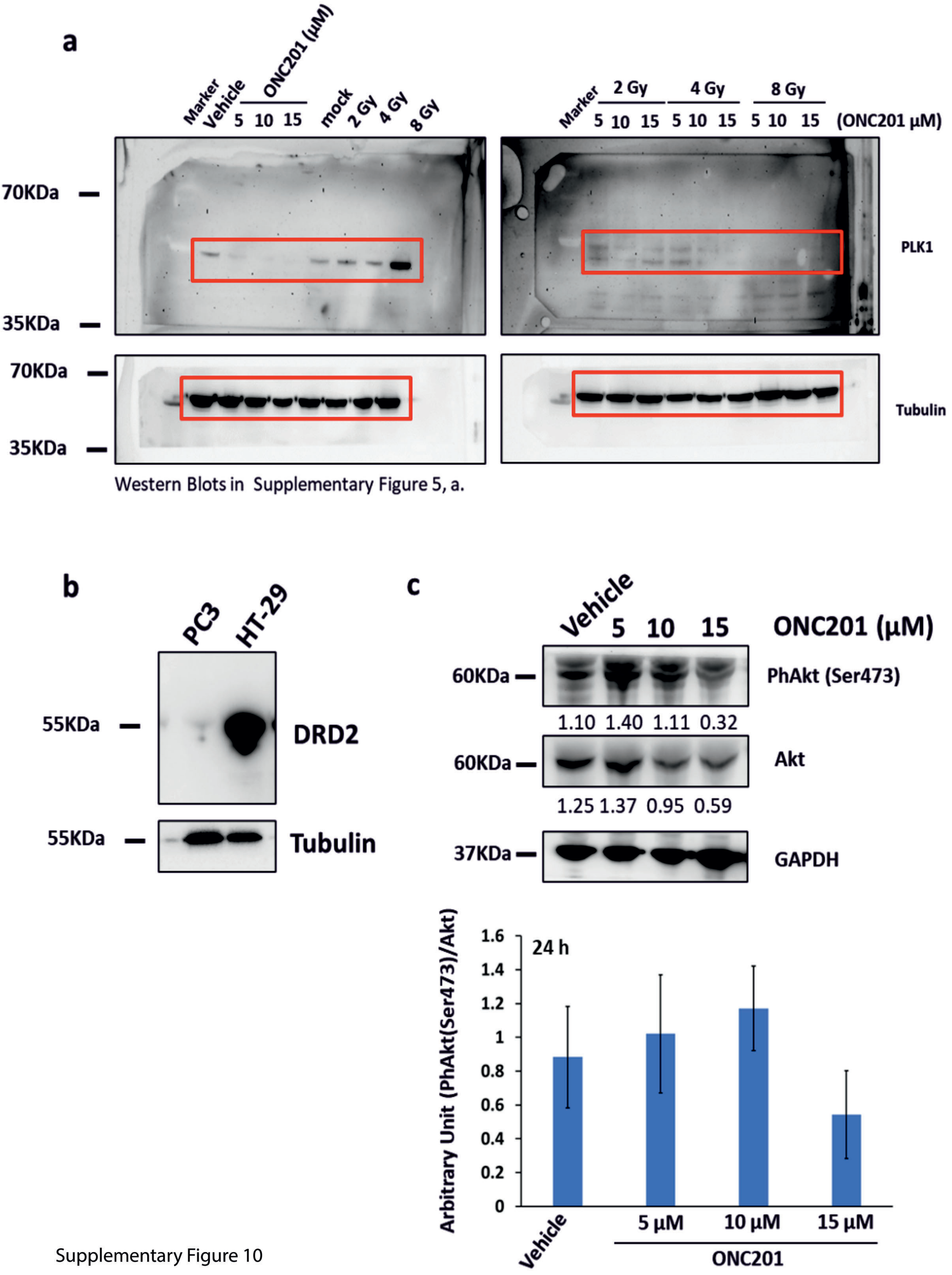

Supplementary Figure 10

a

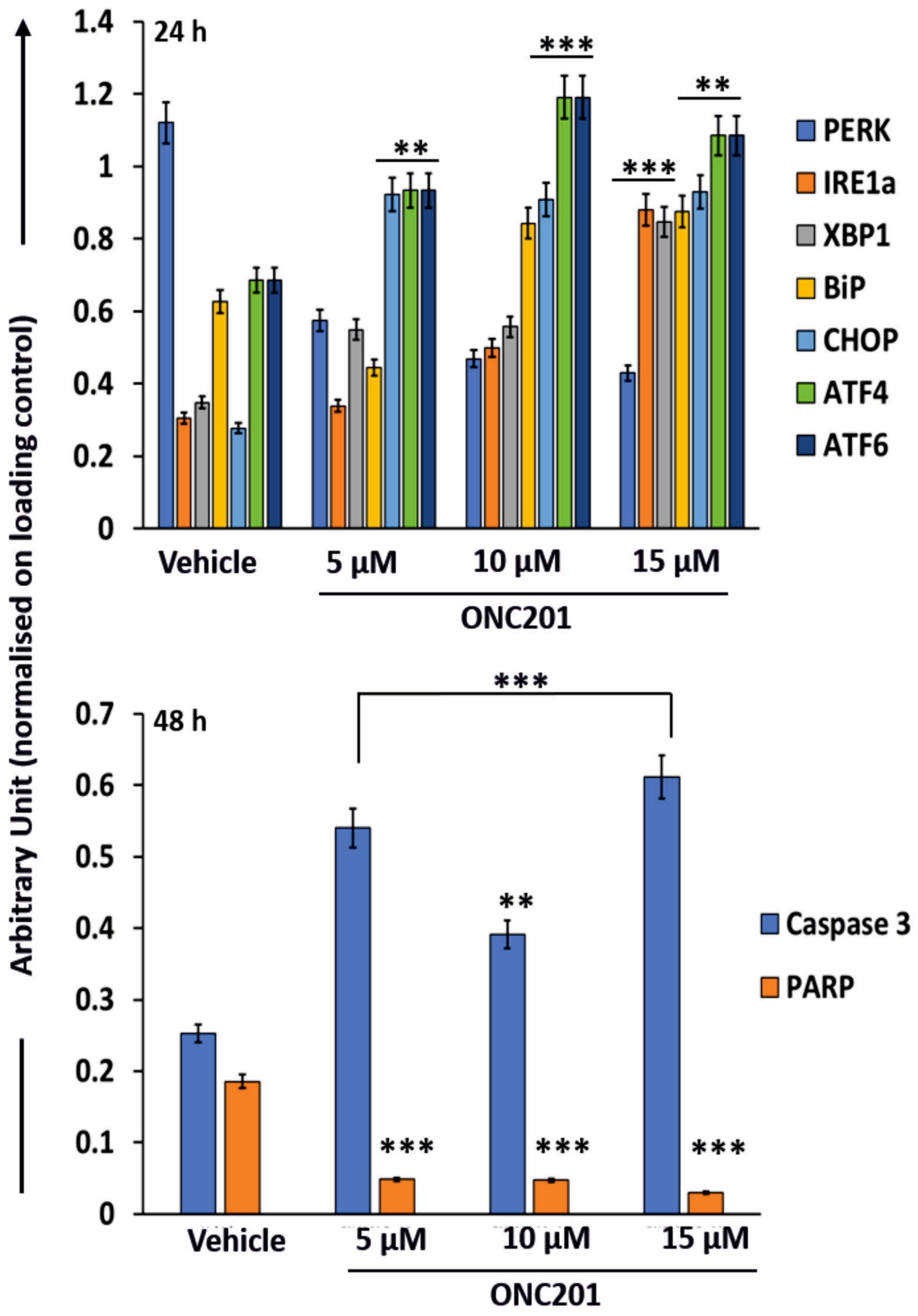

Rappresenative Western Blots in Figure 1, b and e.

b

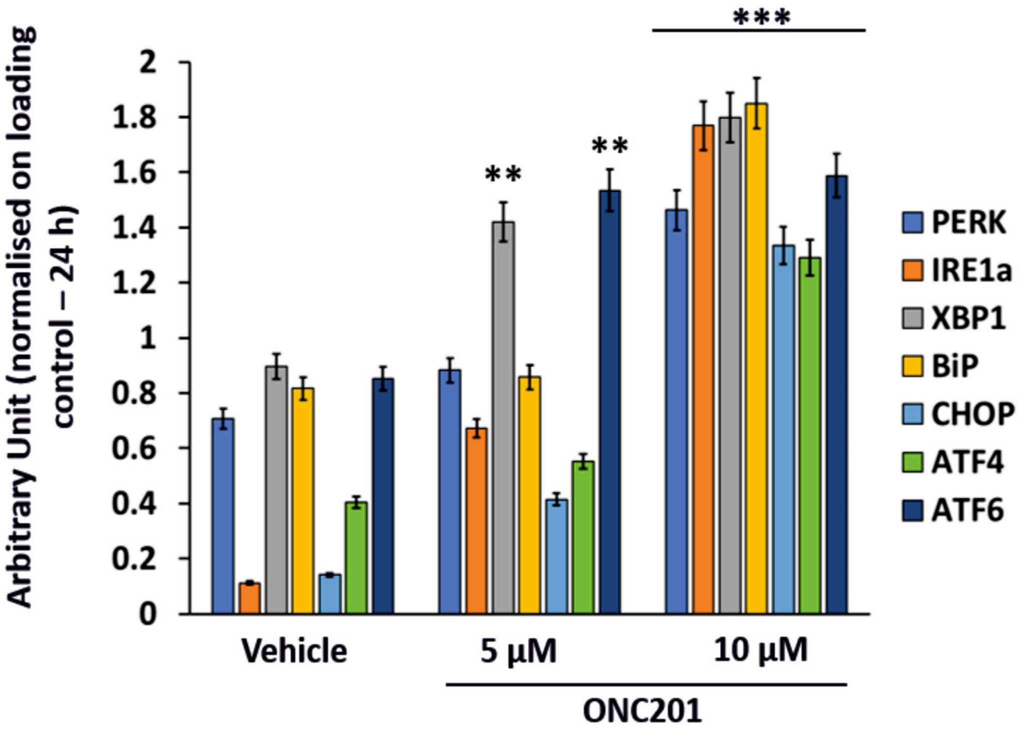

Rappresenative Western Blots in Figure 6, a.

Supplementary Figure 11

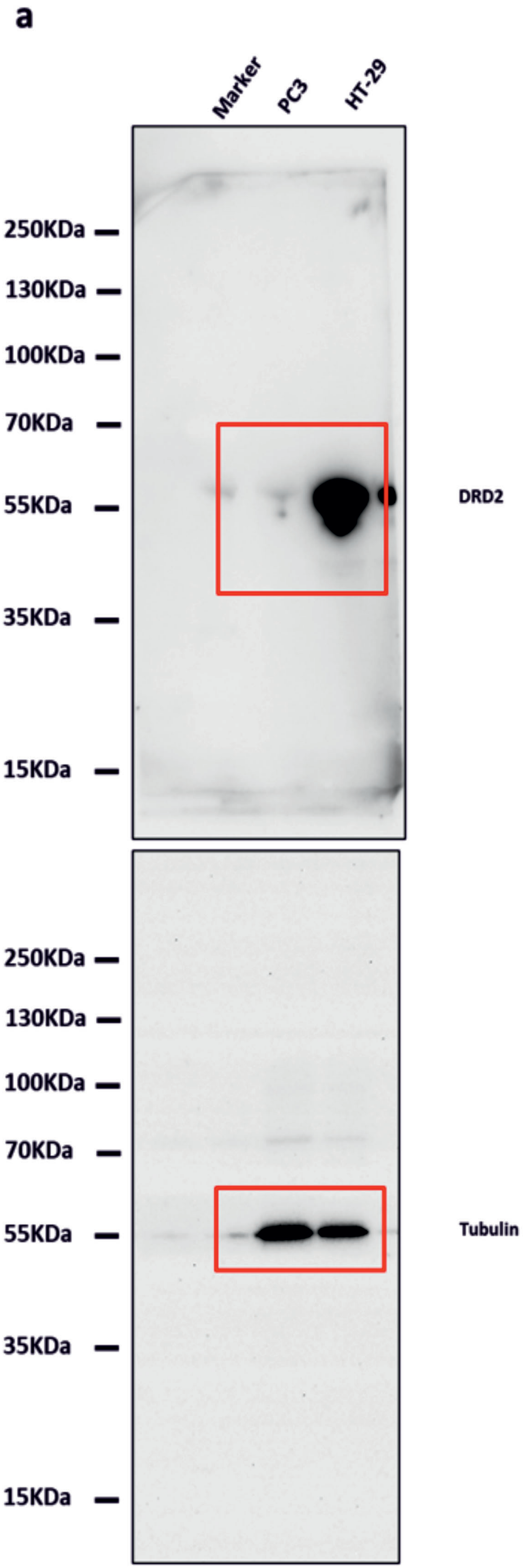

Western Blots in Supplementary Figure 10, b.

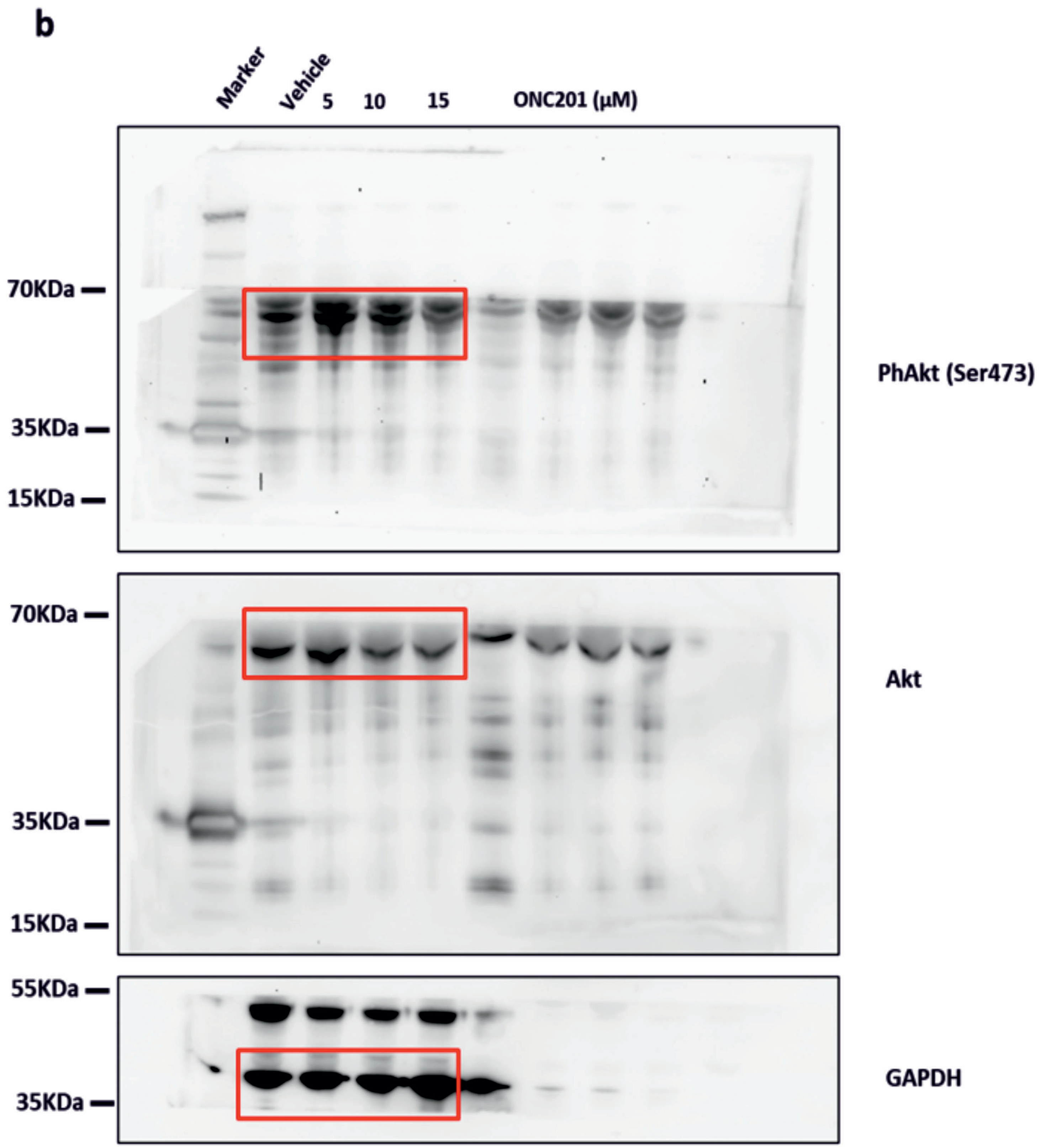

Western Blots in Supplementary Figure 10, c.

Supplementary Figure 12
